# TooManyCellsInteractive: A visualization tool for dynamic exploration of single-cell data

**DOI:** 10.1093/gigascience/giae056

**Published:** 2024-08-22

**Authors:** Conor Klamann, Christie J Lau, Javier Ruiz-Ramírez, Gregory W Schwartz

**Affiliations:** Data Sciences Institute, University of Toronto, Toronto, ON M5G 1Z5, Canada; Princess Margaret Cancer Centre, University Health Network, Toronto, ON M5G 1L7, Canada; Department of Medical Biophysics, University of Toronto, Toronto, ON M5G 1L7, Canada; Princess Margaret Cancer Centre, University Health Network, Toronto, ON M5G 1L7, Canada; Princess Margaret Cancer Centre, University Health Network, Toronto, ON M5G 1L7, Canada; Department of Medical Biophysics, University of Toronto, Toronto, ON M5G 1L7, Canada; Vector Institute, Toronto, ON M5G 1M1, Canada

**Keywords:** single-cell sequencing, data visualization, hierarchical clustering, big data, browser-based, interactive graphical user interface, drug-tolerant persister cells, cell line

## Abstract

**Background:**

As single-cell sequencing technologies continue to advance, the growing volume and complexity of the ensuing data present new analytical challenges. Large cellular populations from single-cell atlases are more difficult to visualize and require extensive processing to identify biologically relevant subpopulations. Managing these workflows is also laborious for technical users and unintuitive for nontechnical users.

**Results:**

We present TooManyCellsInteractive (TMCI), a browser-based JavaScript application for interactive exploration of cell populations. TMCI provides an intuitive interface to visualize and manipulate a radial tree representation of hierarchical cell subpopulations and allows users to easily overlay, filter, and compare biological features at multiple resolutions. Here we describe the software architecture and demonstrate how we used TMCI in a pan-cancer analysis to identify unique survival pathways among drug-tolerant persister cells.

**Conclusions:**

TMCI will facilitate exploration and visualization of large-scale sequencing data in a user-friendly way. TMCI is freely available at https://github.com/schwartzlab-methods/too-many-cells-interactive. An example tree from data within this article is available at https://tmci.schwartzlab.ca/.

## Introduction

Single-cell sequencing quantifies transcriptomic and epigenomic activity at the resolution of individual cells, which enables unprecedented insight into the cellular landscape of biological processes and diseases. However, current approaches for single-cell visualization were not developed to scale with increasingly complex data produced by high-throughput sequencing technologies—both in terms of the number of measured cells and the number of features measured per cell.

A key component of single-cell analysis is to identify distinct cell states and types present within the experimental sample [1–4]. Most standard visualization workflows begin by collapsing the high-dimensional cell features (e.g., genes or chromosome regions) into 2 dimensions using techniques such as principal component analysis (PCA), t-distributed stochastic neighbor embedding (t-SNE), or uniform manifold approximation (UMAP) [5–7]. Current methods apply dimensionality reduction to make the data more amenable for analysis and visualization, a technique that often distorts distances between cells [8–12]. As a result, cells placed closer together on a scatterplot may not necessarily represent cells with higher biological similarity. Dimensionality reduction is commonly followed by unsupervised clustering algorithms such as k-means, Louvain, or Leiden, which are all limited to generating a single-resolution grouping that cannot simultaneously identify subpopulations and is heavily influenced by user-defined parameters [13]. By default, most analysis toolkits also apply clustering on low-dimensional embeddings to reduce computation time, thereby removing potential signals in the data for downstream interpretations. To overcome such limitations, we previously introduced TooManyCells—a suite of tools for cell-clade quantification [1, 2]. The TooManyCells dendrogram depicts all cells starting at the root node, which become recursively bipartitioned at each subsequent child node based on similarity. While TooManyCells preserves distances between cells and presents multiple resolutions of cellular populations, the method produces static representations that generate complex trees with larger datasets.

In addition to methodological limitations of existing visualizations, many bioinformatic tools are out of reach because they require both computational expertise and biological insight for data exploration. To help bridge this gap, interactive tools such as CELLxGENE [14], Cirrocumulus [15], and others [16, 17] facilitate visualization and inquiry of high-throughput single-cell data. More recent tools were designed to assist with specific challenges within analytical workflows such as read alignment [18], parameter selection [19], compute-intensive processes [20], cell-type annotation [21], and lack of familiarity with programming [22, 23]. However, their approaches toward single-cell data analysis remain fundamentally unchanged from that of conventional workflows and do not address scalability issues. Altogether, the limitations of current interactive visualization approaches inevitably affect our ability to interpret the underlying biology and represent a critical issue in high-throughput data analysis.

**Figure 1:**
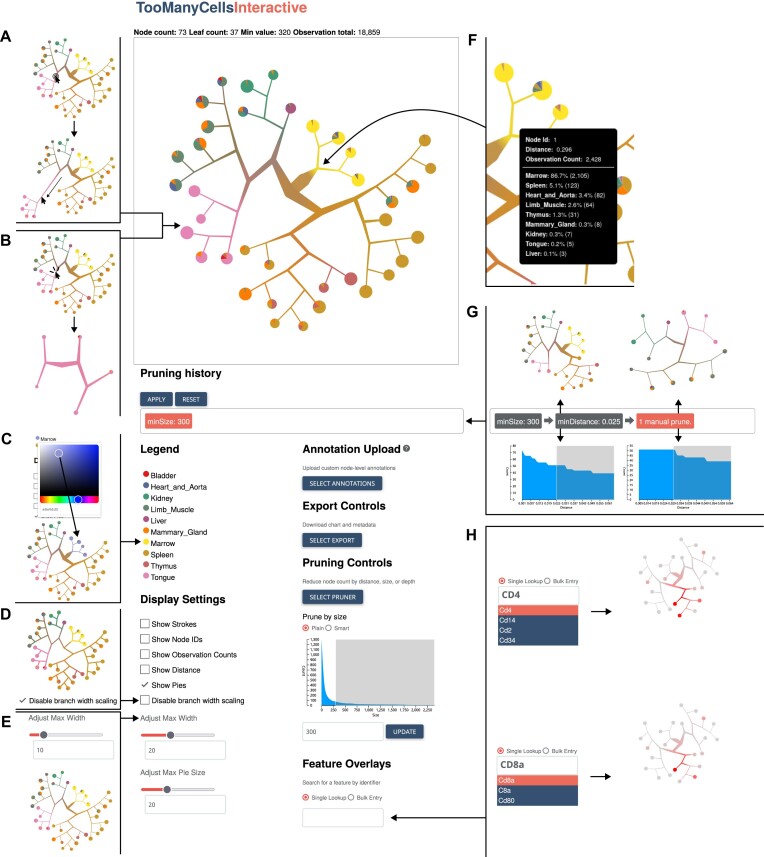
Overview of the TMCI output interface. (A, B) Direct interactions with the main interface. The user may manually edit the tree in the main interface by stretching or shrinking branches (A) or selecting a new tree root (B). (C) Color picker for cell labels through hex value or slider when selecting the label of choice in the legend. (D, E) Visualization features of the tree branches and nodes, including the disabling of branch scaling (D) and adjusting the width of branches (E), among other visualization features. (F) Live-updating tooltips containing statistics for each node. (G) Breadcrumb toolbar containing previous structural changes as the user interactively prunes the tree based on the distribution of nodes. (H) Fuzzy-search bar to see the overlay of a feature on each node in the tree, such as gene expression for each cellular population. The user may select 1 or several features through the fuzzy-search bar and select thresholds for “high” and “low” cutoffs for simultaneous feature overlays (e.g., both *CD4* and *CD8a*).

To address these limitations, we introduce TooManyCellsInteractive (TMCI), a browser-based JavaScript application for interactive exploration of cell populations. TMCI is an easy-to-use tool that displays single-cell data as a radial tree of nested cell clusters and their relationships, and it can be applied to a variety of different data types, including gene expression from single-cell RNA sequencing (scRNA-seq) [1] and chromatin accessibility from single-cell assay for transposase-accessible chromatin (scATAC-seq) [2]. TMCI works seamlessly with TooManyCells dendrograms, so users can interactively explore the tree structure through a responsive dashboard to quickly and easily retrieve population statistics, manually or statistically alter cluster resolution of the tree, quickly overlay feature information, and batch export the display across thousands of trees (Fig. 1). Here, we demonstrate TMCI’s advantages over commonly used visualization tools by benchmarking across several datasets and highlighting an example use case of TMCI to study drug-tolerance mechanisms across multiple cancer types. With an intuitive interface and flexible export system, TMCI is a robust solution to visualize large single-cell datasets. TMCI is open source and packaged with all dependencies at https://github.com/schwartzlab-methods/too-many-cells-interactive. An example tree from data within this article is available at https://tmci.schwartzlab.ca/.

## Results

### Implementation

**Figure 2:**
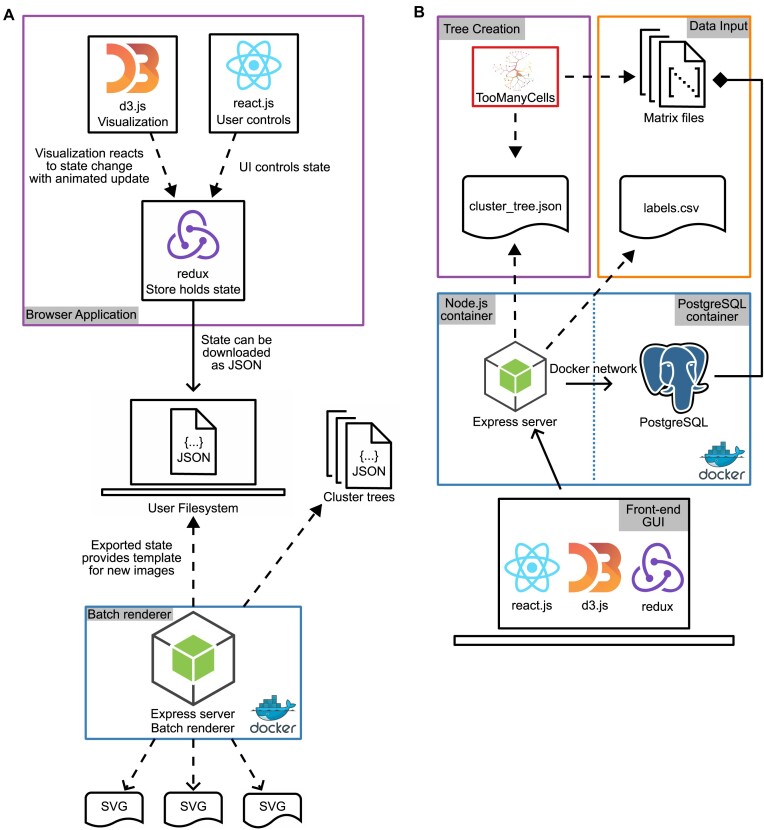
The architecture for TMCI. (A) The front-end architecture of TMCI. The user interacts with the D3 tree visualization and React user interface, which sends state-change requests to Redux. This state-tracking feature enables batch processing: the user may upload a configuration state, which the Express server will read without loading the graphical user interface and automatically export the corresponding SVG. (B) The back-end Express server container takes as input the tree structure and cell label files in the Node application. Similarly, a PostgreSQL container reads the matrix files containing the count matrices with features such as gene expression or chromatin accessibility. The Express container manages feature overlays on the tree through PostgreSQL queries in response to front-end requests. Flowcharts are Unified Modeling Language structured diagrams where dashed closed arrows indicate dependencies, dashed open arrows indicate artifacts, solid closed arrows indicate relationships, and solid diamonds are compositions.

TMCI consists of a browser-based graphical user interface (Fig. 1), a web server, a relational database, a containerized runtime environment, and a collection of initialization and data-processing scripts (Fig. 2). The TMCI browser application is written in TypeScript, a statically typed superset of JavaScript, and implements a variety of frameworks and libraries to provide a highly interactive graphical user interface. Principal UI elements include an interactive radial tree for data visualization and a dashboard-style panel of input controls enabling users to make real-time adjustments to their plots. Such adjustments include node filtering (“pruning”), scale modification, feature overlay, and manual position adjustment (Supplementary Note S1). For saving the tree, TMCI supports image exporting to both PNG and SVG formats.

The browser application’s base architecture is provided by custom React.js components, while state management is handled by Redux and the interactive plots are created with D3.js, a widely used low-level collection of data visualization modules for scaling, event binding, Document Object Model (DOM) traversal, and high-performance animations (Fig. 2).

The back-end Node application transpiles the Typescript to JavaScript using a Webpack bundler and serves it to the user’s browser via an Express application (Fig. 2). If users wish to include custom feature overlays in their plots, such as gene expression data, they may upload the data to the PostgreSQL database that has been configured to connect to the Node server (Supplementary Note S2).

Both the PostgreSQL database and the Node server run in Docker containers, for which TMCI provides a declarative configuration via Docker Compose. TMCI’s containerized architecture allows it to be run on any computer with Docker installed, and the TMCI codebase includes Bash scripts intended as convenience wrappers around commonly used Docker commands that can be easily extended for custom use.

Because D3.js has no strict browser dependencies, TMCI’s radial tree plots can be rendered without a browser interface. TMCI provides both a Node script and a shell script to enable easy programmatic rendering. The scripts require an additional configuration JSON string that can be exported directly from the browser interface. Thus, users may refine their visualizations in the graphical environment and then reuse their configurations as templates for scripted batch processing on the server.

### TMCI reduces time to display trees

**Figure 3:**
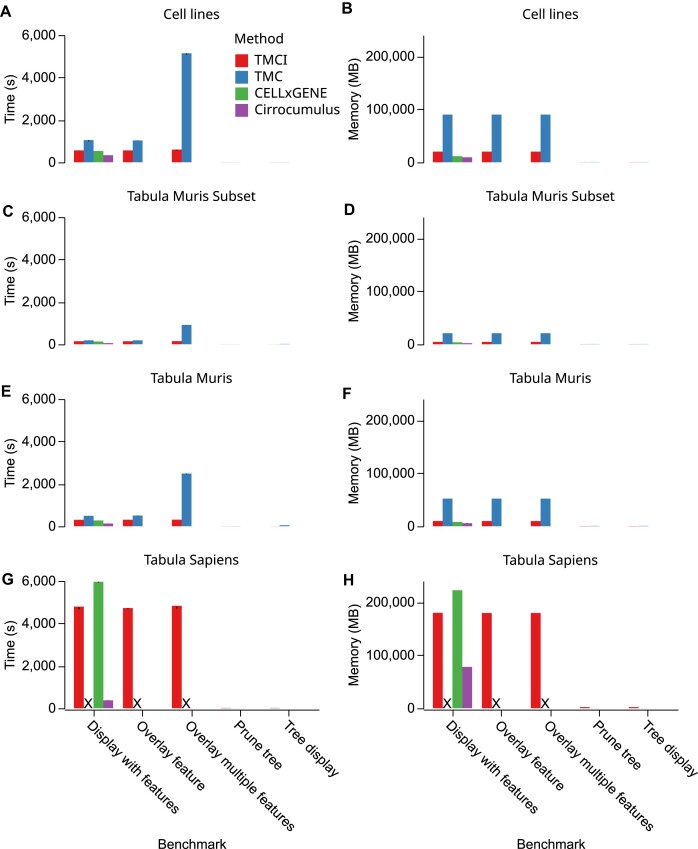
Comparative analysis of performance. (A–F) Comparisons on the *x*-axis including, from left to right, loading a count matrix and displaying a visualization (all programs), overlaying a single feature on a tree (tree programs only), batch processing 5 features on a tree (tree programs only), pruning a larger tree (tree programs only), and rendering a tree without the count matrix loaded (tree programs only). Comparisons were split by 11 samples from 5 cancer cell lines in response to drug treatment (A, B), a set of 10 samples from mouse tissues [24] (C, D), or a set 24 samples from mouse tissues (E, F) and 24 human tissues and organs [25], measuring time (A, C, E, G) or memory usage (B, D, F, H). TMC: TooManyCells; X: Incomplete due to insufficient memory.

To compare the computational time and memory of our TMCI approach to data visualization from both our original, static implementation as well as other commonly used single-cell data exploration tools CELLxGENE [14] and Cirrocumulus [15], we developed 5 benchmarks for common single-cell analyses: loading in all single-cell data and generating a visualization (*display with features*), overlaying colors on the visualization corresponding to a single feature annotation (*overlay feature*), batch processing 5 sequential feature overlays (*overlay multiple features*), adjusting the cluster resolution through tree pruning (i.e., reducing the size of the tree by collapsing child nodes into the parent node; *prune tree*), and rendering the visualization itself without loading the full read matrix (*tree display*). We ran these benchmarks using 54,220 cells from a scRNA-seq dataset of 11 samples across 5 cancer cell lines (Fig. 3A, B), 18,859 cells (“subset”; Fig. 3C, D), and 41,668 cells (Fig. 3E, F) from the Tabula Muris dataset containing 10 mouse organs [24], as well as 483,152 cells from the Tabula Sapiens dataset of 24 tissues and organs from the human body [25].

To assess a baseline performance of each program, we compared the time and memory needed to display trees without feature overlays on our cancer cell line dataset, meaning that no matrix processing was required. Inputs for both programs were the tree and label files generated by TooManyCells, and we ran each benchmark 5 times to account for potential variability. TMCI was 4-fold faster than TooManyCells in the cancer cell line dataset (mean 1.07 vs. 4.62 seconds, *t*-test: $p = 2.74 \times 10^{-18}$; Fig. 3A), demonstrating an order of magnitude speed improvement with our new implementation. Importantly, this upgrade did not come at the cost of memory, as TMCI used $\sim$ 120 MB less memory than TooManyCells (mean 188 vs. 308 MB, *t*-test: $p = 1.87 \times 10^{-14}$; Fig. 3B). As this benchmark did not alter the structure of the tree, we next compared tools by pruning the tree to have nodes containing no fewer than 1,000 cells. This additional processing resulted in TMCI using approximately the same amount of resources as the unpruned tree and TooManyCells, increasing its performance to a mean of 1.64 seconds (*t*-test: $p = 2.39 \times 10^{-14}$) and 300 MB of RAM (*t*-test: $p = 5.56 \times 10^{-16}$; Fig. 3A, B). While the performance increase of TMCI over TooManyCells was consistent across datasets, some gains were 20-fold as with the larger Tabula Muris dataset with insufficient memory to complete the visualization for TooManyCells on the Tabula Sapiens dataset (Fig. 3C–H, Supplementary Tables S1–S4).

Although TMCI outperformed with only tree display and processing, this benchmark did not account for matrix processing. As such, we next compared the performance of each program when rendering feature overlays, which introduces the resource-intensive task of retrieving expression data from the matrix. For a single feature on the cancer cell lines dataset, TMCI outperformed TooManyCells in task duration (mean 554  vs. 1,021 seconds, *t*-test: $p = 1.91 \times 10^{-15}$; Fig. 3A). This advantage remained through TMCI’s greatly reduced memory usage (mean 19.9 vs. 89.8 GB, *t*-test: $p = 2.11 \times 10^{-26}$; Fig. 3B). While this test displayed significant memory gains for TMCI over TooManyCells, a more applicable benchmark is to batch process the creation of several graphics from a single tree with varying gene expression overlays. In this benchmark of 5 features, TMCI outperformed TooManyCells in both time (mean 577 vs. 5,102 seconds, $p = 7.46 \times 10^{-18}$) and memory (mean 19.9 vs. 89.8 GB, $p = 2.44 \times 10^{-33}$; Fig. 3A, B) usage due to the unique persistent feature database, which enables TMCI to generate any number of images after only a single data import operation. TooManyCells, on the other hand, must process the matrix for each new graphic, leading to a linear $\mathcal {O}(n)$ performance where *n* is the number of feature overlays requested. As a result, TMCI was able to generate 10 trees with just 23 seconds longer than a single tree, while TooManyCells took 10 times longer than a single tree. These observations were consistent through all datasets (Fig. 3C–F, Supplementary Tables S1–S4).

To compare with non-tree-based methods, we measured the performance of loading an entire single-cell dataset and producing a visualization. For our cancer cell line dataset, Cirrocumulus was the fastest (mean 331 seconds), with CELLxGENE (mean 528 seconds) and TMCI (mean 550 seconds) close behind, and TooManyCells being the slowest (mean 1 009 seconds; Fig. 3A). Likewise, Cirrocumulus had the lowest memory usage (mean 9.34 GB), followed by CELLxGENE (mean 11.3 GB), TMCI (mean 19.9 GB), and TooManyCells (mean 89.8 GB; Fig. 3B). Importantly, TMCI displays all cluster resolutions, while CELLxGENE and Cirrocumulus only show “flat” clusterings, even though TMCI has a closer performance to these 2 tools than TooManyCells, which is significantly more resource heavy (Supplementary Tables S1 and S2). These observations are consistent in the Tabula Muris datasets but not in the larger Tabula Sapiens, where TMCI has the second lowest time and memory usage, outperforming CELLxGENE (Fig. 3E–H, Supplementary Tables S1–S4). Together, these benchmarks indicate not only the comparable performance of TMCI to a generate static and interactive tree of single-cell data compared to other tools across multiple clustering resolutions but also its ability to quickly and efficiently batch process many trees at once.

### Case study: TMCI effectively delineates subpopulations of cancer drug-tolerant persister cells

**Figure 4:**
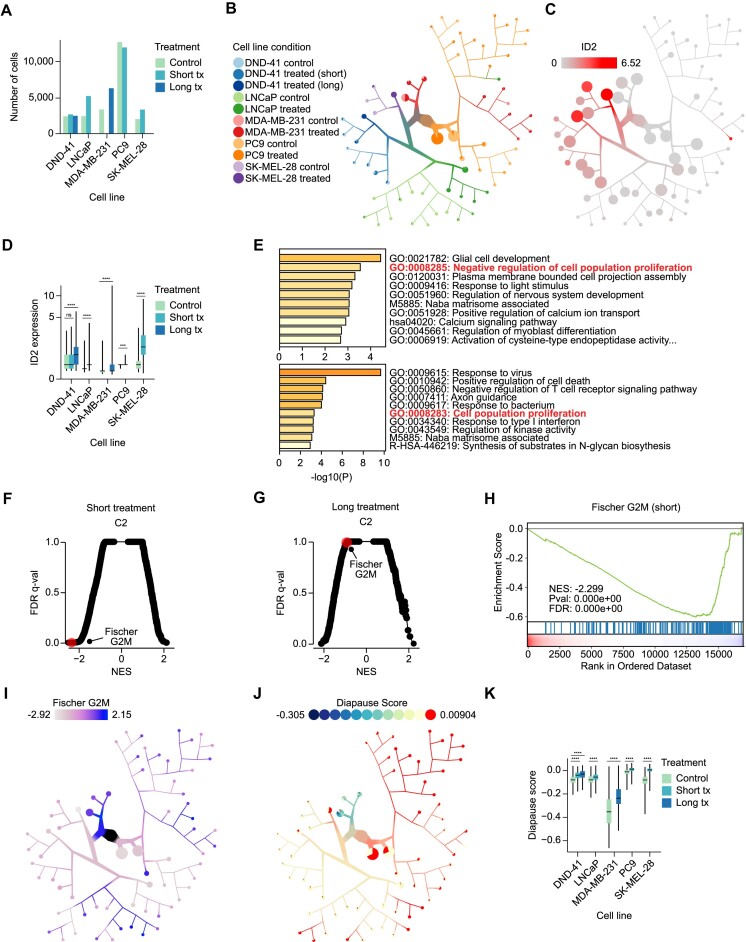
TMCI identifies distinct transcriptional programs across short-term and long-term treated drug-tolerant persister cells across cancer types. (A) Counts of T-cell acute lymphoblastic leukemia (DND-41), pancreatic cancer (LNCaP), melanoma (SK-MEL-28), lung cancer (PC9), and breast cancer (MDA-MB-231) cells from public persister cell scRNA-seq experiments. Each cell line (control) received a short-term (1–3 days) or long-term (6–7 weeks) anticancer treatment. (B, C) TMCI tree of cells from (A) colored by cell line and treatment condition (B) or by average expression of *ID2* per node (C). (D) Box-and-whisker plot of *ID2* expression for cells from each treatment condition. (E) The top 10 enriched pathways determined by Metascape [40] of the top 100 upregulated genes among short-term (top) and long-term (bottom) treated cells. Pathways relevant to the regulation of cellular proliferation are highlighted in red. (F, G) Normalized enrichment scores (NES) and respective *q*-values from gene set enrichment analysis (GSEA) [41] of short-term (F) and long-term (G) treated cells compared to corresponding control cells. “FISCHER_G2_M_CELL_CYCLE” is highlighted in red. (H) GSEA curve of the “FISCHER_G2_M_CELL_CYCLE” gene set for short-term treated cells against untreated cells. (I) NES scores of the “FISCHER_G2_M_CELL_CYCLE” gene set for each node against all other nodes in the TMCI tree from (B). (J) TMCI tree from (B) colored by diapause gene signature. (K) Box-and-whisker of diapause signature scores for cells from each treatment condition. For all box-and-whisker plots, center line: median, box bounds: interquartile range, whiskers: minimum and maximum scores. Statistical annotations represent results of a 1-sided Mann–Whitney *U* test with Benjamini–Hochberg correction. ***$p < {0.001}$, ^****^$p < {0.0001}$, ns: not significant.

To demonstrate the utility of TMCI for quantification and visualization of relationships between diverse single-cell datasets, we explored the transcriptional differences induced by short-term (2–3 days) and long-term (6–7 weeks) treatment of cancer cells *in vitro*. While treatment eliminates the majority of cancer cells, rare populations of drug-tolerant persister cells survive and may potentially act as a reservoir for drug-resistant growth [26]. Persister cells are characterized by a nongenetic, slow-cycling state that is reversible; upon drug holiday, persister cells are resensitized to treatment [27]. We sought to better understand the differences between short- and long-term treatment exposure in these persister cells using TMCI. To this end, we aggregated publicly available scRNA-seq data from 5 independent cancer persister cell experiments across various disease areas and treatment modalities (Fig. 4A, Table 1) [1, 28–31]. The TMCI visualization identified distinct separation between cancer cell lines, followed by division of control and treatment arms (Fig. 4B). This hierarchy suggests that cells of a given cancer type, regardless of drug treatment, are more transcriptionally similar to one another than persister cells across cancer types for most populations.

**Table 1:** Human cancer cell lines from single-cell RNA-sequencing persister cell experiments used in this case study. Corresponding anticancer drugs, treatment duration, and GEO accession numbers are listed

Disease area	Cell line	Treatment	Duration	GEO accession
Prostate cancer	LNCaP	DMSO	48 h	GSM5155455
		Enzalutamide	48 h	GSM5155456
Melanoma	SK-MEL-28	Untreated		GSM4932163
		Dabrafenib	72 h	GSM4932166
Non–small cell lung cancer	PC9	Untreated		GSM3972651
		Erlotinib	72 h	GSM3972652
Breast cancer	MDA-MB-231	Untreated		GSM4684556
		Doxorubicin	7 wk	GSM4684557
T-cell acute lymphoblastic leukemia	DND-41	DMSO	24 h	GSM4121361
		Compound E	24 h	GSM4121362
		Compound E	6 wk	GSM4121364

### TMCI identified differentially expressed *ID2* across persister cell populations

In order to understand how survival programs could be affected by the duration of treatment, we sought to characterize the unique expression profiles among persister cell populations. We identified differentially expressed genes between control and persister cells of each cell line separately and aggregated the complete list of genes using rank product analysis [32]. The batch functionality of TMCI allowed us to efficiently visualize the distribution of top-ranking most differentially expressed genes across the entire dataset collection. From these visualizations, we identified *ID2* as one of the most highly upregulated genes across long-term treated cells in comparison to controls (rank product: 4, permutation test: $p < 2.22 \times 10^{-16}$) but not among the short-term treated cells (rank product: 380, permutation test: $p < 2.22 \times 10^{-16}$) (Fig. 4C, Supplementary Tables S5 and S6). Comparison of *ID2* expression between each control and corresponding treatment arm showed a significant increase of $\log _2$ fold change values for all cell lines (Mann–Whitney *U* test: $p < 0.05$), regardless of treatment duration, with the exception of short-term treated DND-41 cells (Fig. 4D, Supplementary Table S7). *ID2* is known to play a role in tumorigenesis as a key regulator of cell cycle progression and overexpression of *ID2* in cell line experiments modulates proliferative capacity and cell invasiveness [33, 34]. Differential *ID2* expression in our analysis suggests varying proliferative activity between treatment durations.

From the tree structure, we noticed a subset of treated MDA-MB-231 breast cancer cells with particularly high *ID2* expression that did not group together with the predominant cell line cluster (Supplementary Fig. S1a). Rather, this subset in node 4 grouped more closely with PC9 lung cancer cells than with other cells of the same disease type and treatment condition in node 126. To explore the differences underlying this distinct cell state, we performed differential expression analysis comparing treated MDA-MB-231 cells of node 4 against node 126 (Supplementary Table S8). Metascape analysis of the top 100 most downregulated genes identified “negative regulation of cell differentiation” (hypergeometric test: $p = 4.68 \times 10^{-4}$) and “PTEN regulation” (hypergeometric test: $p = 9.55 \times 10^{-3}$) as significantly enriched pathways (Supplementary Fig. S1b). We corroborated these results through gene set enrichment analysis, which identified signals of dysregulated *ID2, KRAS, PTEN*, and *YAP1* expression among the most differentially represented oncogenic signatures (Supplementary Table S9). Interestingly, many of these signatures were derived from RNA interference screens of *KRAS*G13D-mutant cell lines for synthetic lethal targets [35]. MDA-MB-231 cells also harbor this oncogenic mutation, which drives constitutive signaling of the KRAS^G13D^ protein [36, 37]. However, we found significant downregulation of *KRAS* expression and underrepresentation of its target genes, accompanied by upregulation of *KRAS*-mutant synthetic lethal vulnerabilities *TBK1, YAP1*, and *STK33* within the subset of interest [38, 39] (Mann–Whitney *U* tests: *KRAS*  $\log _2$FC $-$1.80, $p = 1.41 \times 10^{-36}$; *TBK1*  $\log _2$FC 0.682, $p = {0.0320}$; *YAP1*$\log _2$FC 0.932, $p = 1.21 \times 10^{-7}$; *STK33*  $\log _2$FC 1.312, $p = {0.700}$; Supplementary Table S8, Supplementary Fig. S1c). Altogether, these findings suggest multiple treated populations, one of which undergoes activation of *KRAS*-mutant compensatory signaling within a subset of treated MDA-MB-231 cells, and demonstrate some of the advantages of a tree-based approach for single-cell analysis.

### TMCI identifies distinct proliferation mechanisms within persister cell populations

To interrogate the ongoing biological mechanisms within short- and long-term treated persister cell populations, we performed pathway analysis using the top 100 upregulated differentially expressed genes in the treated cells. Metascape [40] analysis of the differentially expressed genes from short-term treated cells identified “negative regulation of cell population proliferation” as a key biological process (hypergeometric test: $p = 2.88 \times 10^{-4}$; Fig. 4E). Conversely, the same analysis performed on differentially expressed genes identified “cell population proliferation” enrichment in long-term treated cells, suggesting an increase of cellular proliferation across pathways (hypergeometric test: $p = 4.89 \times 10^{-4}$; Fig. 4E). Subsequent exploration of the full list of differentially expressed genes using gene set enrichment analysis [41] returned markedly distinct biological programs between the short- and long-term treated populations. Among short-term treated populations, the most significantly decreased hits were found to be associated with various proliferation and cell cycle regulation programs. In line with our previous findings, these programs were not significantly downregulated among long-term treated cells (Supplementary Table S10). Among these gene sets, we found the expression of “FISCHER_G2_M_CELL_CYCLE” significantly decreased among short-term treated cells (NES $= -2.30$, Kolmogorov–Smirnov test: $p < 2.22 \times 10^{-16}$) but not among long-term treated cells (NES $= -0.891$, Kolmogorov–Smirnov test: $p = 0.747$; Fig. 4F–I, Supplementary Fig. S2a). Consistent with this observation, additional G2M checkpoint and E2F target gene sets showed similar patterns (Supplementary Table S11). These findings suggest that persister cells utilize distinct pathways associated with modulation of proliferation and cell cycling throughout the duration of treatment.

### TMCI identifies subpopulations with highly expressed diapause programs

As we identified proliferation and cell cycle factors associated with treatment duration, we were interested in understanding the temporal expression of diapause programs within the various persister cell populations. Diapause is a reversible state of suspended embryonic development triggered by adverse environmental conditions [42]. Similarly, persister cells that survive throughout exposure to treatment undergo transcriptional adaptations resembling a diapause-like state [43, 44]. Overlaying diapause gene signature scores on the tree structure showed enrichment in all treated subpopulations compared to controls (Fig. 4J, Supplementary Fig. S2b).

Comparison between each control and treatment arm showed significantly increased diapause signature scores in all treated cell lines, again regardless of treatment duration (Mann–Whitney *U* test: $p < 0.05$; Fig. 4K, Supplementary Fig. S2c–f, Supplementary Table S12). For DND-41, which includes measurements of both short- and long-term treatment durations, the median diapause signature score increased from control to short term to long term, suggesting a direct correlation between diapause gene signature scores and treatment duration. Confirming that the easily seen difference in diapause signature scores within each cell line was significant, we compared the TMCI visualization against a traditional scatterplot generated with CELLxGENE (Supplementary Fig. S2g–h, Supplementary Fig. S3, Supplementary Note S4). Although TMCI and CELLxGENE had the same diapause signature scores, the significantly different subpopulations were more easily seen in TMCI’s tree. Together, our analysis points to persister cells with different proliferation activity depending on treatment duration.

## Discussion

As high-throughput single-cell technologies continue to measure increasing numbers of cells, we need new visualization tools to better identify and interpret cell states. Here we present TMCI as a powerful, interactive solution that simplifies data exploration of large datasets. These visualizations are intuitive, supporting easy tree manipulation through statistical or manual pruning, color mapping, feature overlays, and more. With these features, identification of rare cellular populations is straightforward compared to previous iterations of single-cell data figures. Importantly, these benefits are not at the cost of performance, with TMCI either outperforming or on par with alternative interactive visualizations. As we implemented TMCI as a web server, users can easily and quickly access large datasets with little computational impact on their local host. As a result of TMCI’s speed, its batch-processing capability allows for quick plotting of thousands of trees derived from a single, manually customized tree.

Using the numerous features afforded by TMCI, we delineated cellular populations from drug-treated cancer cell lines and identified distinct transcriptional programs between short- and long-term treated cell lines. These programs included cell proliferation pathways downregulated in short-term persister cell states, which are then subsequently lost in the long-term cellular populations across all cancer types measured. This finding extended to the diapause signature, which was increased in persister cells, in concordance with previous studies, but here across cancer type. Together, TMCI identified transcriptional programs that are dependent on treatment duration, suggesting further investigation on the timing of treatment for persister cells.

Although TMCI is a feature-rich application for tree structure exploration, several future directions could enhance TMCI’s capabilities. While projection-based visualizations such as t-SNE and UMAP have limited capabilities in identifying cell relationships, they are still widely used among the single-cell sequencing analysis community. To link these visualizations together, a new user interface could be created for simultaneous investigation similar to Sleepwalk [9]. These combinations of multiple embeddings may also include other dashboard features such as gene expression heatmaps and enriched pathways. Furthermore, TMCI currently displays relationships generated from a single data modality. As new multiomic technologies sequence both RNA and chromatin accessibility or protein from the same cell, there exists new opportunities for TMCI to integrate multiple data modalities in a tree structure. In the meantime, through our application to drug-treated cancer cell lines, we show that big data visualization tools will be necessary as available data grow, and we provide TMCI as a solution for visualizing tree-based relationships in such data.

## Materials and Methods

### Benchmarks

We performed benchmarks using an AWS EC2 instance running Ubuntu 20.04 and Docker 20.10.17 with 64x Intel Xeon Platinum 8375C CPU @ 2.90 GHz and 534 GB RAM. We compared CELLxGENE [14], Cirrocumulus [15], TooManyCells [1, 2], and TMCI using 54,220 cells from 5 cancer cell lines, 41,668 cells from the Tabula Muris dataset [24], a smaller subset of 18,859 cells from the Tabula Muris dataset, and 483,152 cells from the Tabula Sapiens dataset [25]. For each method, we devised 5 benchmarks for compute time and memory usage, some of which were unique to tree-based approaches. For all methods, we loaded all single-cell data and ran the default options to generate visualizations (*display with features*). Based on this benchmark, we also overlaid a single color on the visualization corresponding to a single feature annotation (*overlay feature*) or also used batch processing for 5 sequential feature overlays (*overlay multiple features*). Specific to tree-based approaches, we measured tree pruning by collapsing child nodes into the parent node (*prune tree*). We also benchmarked performance when only displaying the full tree visualization without loading the entire single-cell matrix (*tree display*). We ran each benchmark 5 times to account for variability in processing time and memory.

### Preprocessing of drug-treated cancer scRNA-seq data

To demonstrate the utility of TMCI, we investigated drug-tolerant persister cell populations, which are capable of surviving anticancer drug treatment through nongenetic programming of reversible mechanisms [27]. We aggregated publicly available scRNA-seq data from 5 *in vitro* persister experiments, including prostate cancer, melanoma, non–small cell lung cancer, breast cancer, and T-cell acute lymphoblastic leukemia cell lines (Table 1). The duration of anticancer drug treatment for each cell line varied from short term (2–3 days) to long term (6–7 weeks), enabling the identification of persister cells across cancer types and time. All datasets were previously generated using similar library preparation methods (10x Genomics 3′ Single Cell Gene Expression), sequencing platforms (Illumina), and alignment pipelines (Cell Ranger). After manual checks to verify that the files contained raw read count data, we aggregated the matrices using AnnData and Scanpy [4] tools in Python. We applied all normalization and filtering using the TooManyCells command-line tool, based on its original default parameters of term frequency-inverse document frequency (TF-IDF) normalization and filtering for cells expressing at least 250 transcripts and genes detected in at least 1 cell [1]. For other batch-effect correction techniques such as Harmony [45], we recommend using our TooManyCells (à la Python) Python implementation, which better handles noncount, transformed embeddings and is fully compatible with Scanpy [4] (Supplementary Note S3).

### Generating drug-treated cancer cell trees

After data normalization and filtering, we used TooManyCells to generate a tree and identify transcriptionally distinct subpopulations within our dataset [1]. In brief, TooManyCells implements a matrix-free hierarchical spectral clustering approach [46] to recursively partition scRNA-seq cell data into similar groups, and it uses Newman–Girvan modularity [47] as an indicator for reaching a leaf in the tree. The resulting tree structure depicts all cells at the central root node, with subdividing branches for each group partition until any additional split would be considered random. This information is encoded in the cluster_tree.json output file and can be viewed interactively through TMCI. We used the resulting tree structure groupings as input for TMCI, through which we applied minimum distance search pruning at a cutoff of 0.019 to improve the visibility of small subpopulations.

### Measuring differential expression across cellular populations

Using the tree structure, we conducted differential gene expression analysis between control and persister cell states of each cell individually, using the TooManyCells “differential” functionality with upper quartile normalized read counts. From the resulting $\log _2$ fold change values, we aggregated a list of differentially expressed genes across cell lines using rank product analysis [32]. These results identified genes that are more broadly associated with the persister state across drug treatments and cancer disease types. We used the batch functionality of TMCI to iterate through the list of top-ranking gene targets and visually identified *ID2* as a potential target of interest on account of its high expression among long-term treated cell lines, which we did not observe across short-term treated persister cells. These findings corroborated with statistical comparisons of expression between treatment conditions within each given cell line, highlighting *ID2* as a target of interest.

To explore the biological mechanisms associated with each cell state, we conducted gene set enrichment analysis [41] across the tree structure. For this analysis, we calculated the $\log _2$ fold change values of each node against all other cells using the methods from Scanpy “rank_genes_groups.” With each ordered gene list, we ran the GSEApy “preranked” module with MSigDB Hallmark, C2 (curated), and C6 (oncogenic) gene sets [48]. We used 2-sided statistical tests for all analyses.

## Availability of Supporting Source Code and Requirements

1. **Project name:** TooManyCellsInteractive

 **Project homepage:**  https://github.com/schwartzlab-methods/too-many-cells-interactive

 **Operating system:** Platform independent

 **Programming language:** TypeScript, JavaScript

 **Other requirements:** Docker, Docker-compose

 **License:** GNU General Public License v3.0

 RRID:SCR_025315

 **Note:** Archival versions of the code are available via Software Heritage [49] and figshare [50], with a tutorial at https://schwartzlab-methods.github.io/too-many-cells-interactive/.

2. **Project name:** TooManyCells (à la Python)

 **Project homepage:**  https://github.com/schwartzlab-methods/too-many-cells-python

 **Operating system:** Platform independent

 **Programming language:** Python

 **Other requirements:** Graphviz (https://graphviz.org/)

 **License:** GNU Affero General Public License v3.0

 **PyPi:** toomanycells (https://pypi.org/project/toomanycells/)

 RRID:SCR_025327

## Supplementary Material

giae056_GIGA-D-23-00386_Original_Submission

giae056_GIGA-D-23-00386_Revision_1

giae056_GIGA-D-23-00386_Revision_2

giae056_Response_to_Reviewer_Comments_Original_Submission

giae056_Response_to_Reviewer_Comments_Revision_1

giae056_Reviewer_1_Report_Original_Submission Qingnan Liang, Ph.D. -- 1/26/2024 Reviewed

giae056_Reviewer_2_Report_Original_Submission Mehmet Tekman -- 2/12/2024 Reviewed

giae056_Reviewer_3_Report_Original_Submission Georgios Fotakis -- 2/19/2024 Reviewed

giae056_Reviewer_3_Report_Revision_1 Georgios Fotakis -- 6/10/2024 Reviewed

giae056_Supplemental_Files

## Data Availability

The GEO accession numbers for each dataset reported in this article are GSM5155455 and GSM5155456 (prostate cancer), GSM4932163 and GSM4932166 (melanoma), GSM3972651 and GSM3972652 (non–small cell lung cancer), GSM4684556 and GSM4684557 (breast cancer), and GSM4121361, GSM4121362, and GSM4121364 (T-cell acute lymphoblastic leukemia). Archival versions of the code are available via Software Heritage [49] and figshare [50]. Code for analyses within this article is available at https://github.com/schwartzlab-methods/too-many-cells-interactive-paper-analyses.

## Additional Files

**Supplementary Fig. S1**. Treated MDA-MB-231 breast cancer cells cluster into distinct cell states. (a) The TMCI tree from Fig. 4B with node identifiers (left) with a collapsed focus on MDA-MB-231 (top right). A subset of treated MDA-MB-231 breast cancer cells clustered together into node 4, separately from the predominant cluster in node 126 and containing a subgroup with high *ID2* expression in node 48 (bottom right). (b) The top 4 enriched pathways determined from Metascape analysis of the 100 most downregulated genes. Pathways corroborating our findings from gene set enrichment analysis are highlighted in red. (c) Box-and-whisker plot of expression of *KRAS* and known *KRAS*-mutant synthetic lethal partners. For all box-and-whisker plots, center line: median, box bounds: interquartile range, whiskers: minimum and maximum scores. Statistical annotations represent results of a 1-sided Mann–Whitney *U* test with Benjamini–Hochberg correction. ns: not significant, *$p < {0.05}$, ****$p < {0.0001}$.

**Supplementary Fig. S2**. Short-term treatment across cell lines has lower proliferative enrichment. (a) Gene set enrichment analysis (GSEA) curve for the “FISCHER_G2_M_CELL_CYCLE” gene set in long-term treated cells. (b) TMCI tree from Fig. 4B colored by diapause signature score per node cluster (red: greater than 1 median absolute deviation away from the median diapause signature score; gray: otherwise). (c) GSEA curves of the upregulated diapause signature score gene set from short-term (left) or long-term (right) treated cells. (d) TMCI tree from Fig. 4B colored by normalized enrichment scores of the upregulated diapause gene set comparing each node to every other node. (e) GSEA curves of the downregulated diapause signature score gene set as in (c). (f) Normalized enrichment scores for the downregulated diapause signature score gene set as in (d). (g, h) CELLxGENE UMAP scatterplots of the same data as in (b) colored by cell line (g) or diapause signature scores (h) per cell.

**Supplementary Fig. S3**. Excerpt from Fig. 4B (a) and Supplementary Fig. S2g (b) for cell line annotations and Fig. 4J (c) and Supplementary Fig. S2h (d) for diapause score overlays for cell line data visualized using TMCI (a, c) and CELLxGENE (b, d) for a side-by-side comparison. (e) CELLxGENE visualization colored by cluster annotations to more directly compare with tree clusters from TMCI.

**Supplementary Fig. S4**. (a, b) Visualizations of read count data after Harmony [45] batch-effect correction for cell line, within each treatment condition. We used the resulting PCA embeddings to generate UMAP embeddings, displayed in CELLxGENE (a), and TooManyCells tree structure, displayed in TMCI (b). We set TooManyCells parameters for corrected data according to recommended defaults (Supplementary Note S3). We pruned the tree with “plain distance search” of 0.05 to improve visual clarity of the clusters.

**Supplementary Table S1**. Mean execution time for each performance benchmark across cells from 10 mouse tissues [24] ($n = {18\, 859}$ cells), 24 mouse tissues ($n = 41\, 688$ cells), 24 human tissues and organs ($n = 483\, 152$ cells) [25], or 11 samples from 5 cancer cell lines in response to drug treatment ($n = {54\, 220}$ cells).

**Supplementary Table S2**. Pairwise *t*-test results for each execution time performance benchmark across datasets from Supplementary Table S1. Benjamini–Hochberg method was used for testing and adjustment of *p*-values.

**Supplementary Table S3**. Mean memory usage for each performance benchmark across datasets from Supplementary Table S1.

**Supplementary Table S4**. Pairwise *t*-test results for each memory usage performance benchmark across datasets from Supplementary Table S1. Benjamini–Hochberg method was used for testing and adjustment of *p*-values.

**Supplementary Table S5**. Rank product of differentially expressed genes between long-term treated cells ($n = {8\, 654}{}$) and their corresponding controls ($n = {5\, 639}{}$). Mann–Whitney *U* test was used to calculate *p*-values.

**Supplementary Table S6**. Rank product of differentially expressed genes between short-term treated cells ($n = {22\, 965}{}$) and their corresponding controls ($n = {16\, 962}{}$). Mann–Whitney *U* test was used to calculate *p*-values.

**Supplementary Table S7**. $\log _2{\mbox{fold change}}$ of upper quartile-normalized gene expression between treated cells and their corresponding controls within a given cell line. Mann–Whitney *U* test was used to calculate *p*-values, and Benjamini–Hochberg method was used for testing and adjustment of *p*-values.

**Supplementary Table S8**. $\log _2{\mbox{fold change}}$ of log-normalized gene expression between a subset of treated MDA-MB-231 cells at node 4 and the predominant cluster at node 126. Mann–Whitney *U* test was used to calculate *p*-values, and Benjamini–Hochberg method was used for testing and adjustment of *p*-values.

**Supplementary Table S9**. Gene set enrichment analysis results for treated MDA-MB-231 cells at node 4 and node of upper quartile-normalized gene expression between treated cells and their corresponding controls within a given cell line. Mann–Whitney *U* test was used to calculate *p*-values, and Benjamini–Hochberg method was used for testing and adjustment of *p*-values.

**Supplementary Table S10**. Gene set enrichment analysis results for long-term treated cells ($n = {8\, 654}{}$) in comparison to their corresponding controls ($n = {5\, 639}{}$), using the Hallmark, C2, and C6 gene sets.

**Supplementary Table S11**. Gene set enrichment analysis results for short-term treated cells ($n = {22\, 965}{}$) in comparison to their corresponding controls ($n = {16\, 962}{}$), using the Hallmark, C2, and C6 gene sets.

**Supplementary Table S12**. Comparison of diapause scores between treated cells and their corresponding control conditions within a given cell line. One-sided Mann–Whitney *U* test was used to evaluate higher diapause scores among treated conditions compared to control. Benjamini–Hochberg method was used for testing and adjustment of *p*-values.

## Abbreviations

GSEA: gene set enrichment analysis; NES: normalized enrichment score; PCA: principal component analysis; RAM: random-access memory; scATAC-seq: single-cell assay for transposase-accessible chromatin; scRNA-seq: single-cell RNA sequencing; TMC: TooManyCells; TMCI: TooManyCellsInteractive; t-SNE: t-distributed stochastic neighbor embedding; UMAP: uniform manifold approximation and projection; DOM: Document Object Model.
