# Supplementary material for: TooManyCellsInteractive: A visualization tool for dynamic exploration of single-cell data
Source: Gigascience. 2024 Aug 22;13:giae056. doi: 10.1093/gigascience/giae056 (PMC11340645; doi:10.1093/gigascience/giae056)
Supplement: giae056_GIGA-D-23-00386_Revision_1 [file giae056_giga-d-23-00386_revision_1.pdf]

GigaScience

TooManyCellsInteractive: a visualization tool for dynamic exploration of single-cell data

--Manuscript Draft--

**Manuscript Number:**	GIGA-D-23-00386R1
**Full Title:**	TooManyCellsInteractive: a visualization tool for dynamic exploration of single-cell data
**Article Type:**	Technical Note

Funding Information:		
	University of Toronto (Data Sciences Institute Research Software Development Support Program)	Dr. Gregory Schwartz
	Canadian Cancer Society (707484)	Dr. Gregory Schwartz
	Natural Sciences and Engineering Research Council of Canada (RGPIN-2023-04713)	Dr. Gregory Schwartz
	Natural Sciences and Engineering Research Council of Canada (DGECR-2023-00395)	Dr. Gregory Schwartz
	Social Sciences and Humanities Research Council (NFRFE-2022-00681)	Dr. Gregory Schwartz
	Canada Research Chairs Program	Dr. Gregory Schwartz
	Princess Margaret Cancer Foundation	Dr. Gregory Schwartz
	University of Toronto (University of Toronto Data Sciences Institute Doctoral Student Fellowship)	Ms. Christie Lau

Abstract:

Background: As single-cell sequencing technologies continue to advance, the growing volume and complexity of the ensuing data present new analytical challenges. Large cellular populations from single-cell atlases are more difficult to visualize and require extensive processing to identify biologically relevant subpopulations. Managing these workflows is also laborious for technical users and unintuitive for non-technical users.
Results: We present TooManyCellsInteractive (TMCI), a browser-based JavaScript application for interactive exploration of cell populations. TMCI provides an intuitive interface to visualize and manipulate a radial tree representation of hierarchical cell subpopulations and allows users to easily overlay, filter, and compare biological features at multiple resolutions. Here we describe the software architecture and demonstrate how we used TMCI in a pan-cancer analysis to identify unique survival pathways among drug-tolerant persister cells.
Conclusions: TMCI will facilitate exploration and visualization of large scale sequencing data in a user-friendly way. TMCI is freely available at https://github.com/schwartzlab-methods/too-many-cells-interactive. An example tree from data within this paper is available at https://tmci.schwartzlab.ca/.

**Corresponding Author:**	Gregory Schwartz<br>University Health Network<br>Toronto, CANADA
**Corresponding Author Secondary Information:**	
**Corresponding Author's Institution:**	University Health Network
**Corresponding Author's Secondary Institution:**	
**First Author:**	Conor Klamann
**First Author Secondary Information:**	
**Order of Authors:**	Conor Klamann
	Christie Lau

Powered by Editorial Manager® and ProduXion Manager® from Aries Systems Corporation

	Javier Ruiz-Ramírez
	Gregory Schwartz
**Order of Authors Secondary Information:**	
**Response to Reviewers:**	Please see `response_to_reviewers.pdf` for a complete point-by-point response.
**Additional Information:**	
**Question**	**Response**
Are you submitting this manuscript to a special series or article collection?	No
**Experimental design and statistics**<br><br>Full details of the experimental design and statistical methods used should be given in the Methods section, as detailed in our Minimum Standards Reporting Checklist. Information essential to interpreting the data presented should be made available in the figure legends.<br><br>Have you included all the information requested in your manuscript?	Yes
**Resources**<br><br>A description of all resources used, including antibodies, cell lines, animals and software tools, with enough information to allow them to be uniquely identified, should be included in the Methods section. Authors are strongly encouraged to cite Research Resource Identifiers (RRIDs) for antibodies, model organisms and tools, where possible.<br><br>Have you included the information requested as detailed in our Minimum Standards Reporting Checklist?	Yes
**Availability of data and materials**<br><br>All datasets and code on which the conclusions of the paper rely must be either included in your submission or deposited in publicly available repositories (where available and ethically	Yes

Powered by Editorial Manager® and ProduXion Manager® from Aries Systems Corporation

appropriate), referencing such data using a unique identifier in the references and in the “Availability of Data and Materials” section of your manuscript.<br><br>Have you have met the above requirement as detailed in our Minimum Standards Reporting Checklist?	

Manuscript Click here to access/download;Manuscript;manuscript.pdf

TooManyCellsInteractive: a visualization tool for dynamic exploration of single-cell data

Conor Klamann[1,*], Christie J. Lau[2-3,*], Javier Ruiz-Ramírez[2], and Gregory W. Schwartz[2-4]

[1]Data Sciences Institute, University of Toronto, Toronto, ON M5G 1Z5, Canada

[2]Princess Margaret Cancer Centre, University Health Network, Toronto, ON M5G 1L7, Canada

[3]Department of Medical Biophysics, University of Toronto, Toronto, ON M5G 1L7, Canada

[4]Vector Institute, Toronto, ON M5G 1M1, Canada

email:gregory.schwartz@uhn.ca

[*]These authors contributed equally to this work

Abstract

Background: As single-cell sequencing technologies continue to advance, the growing volume and complexity of the ensuing data present new analytical challenges. Large cellular populations from single-cell atlases are more difficult to visualize and require extensive processing to identify biologically relevant subpopulations. Managing these workflows is also laborious for technical users and unintuitive for non-technical users.
Results: We present TooManyCellsInteractive (TMCI), a browser-based JavaScript application for interactive exploration of cell populations. TMCI provides an intuitive interface to visualize and manipulate a radial tree representation of hierarchical cell subpopulations and allows users to easily overlay, filter, and compare biological features at multiple resolutions. Here we describe the software architecture and demonstrate how we used TMCI in a pan-cancer analysis to identify unique survival pathways among drug-tolerant persister cells.
Conclusions: TMCI will facilitate exploration and visualization of large scale sequencing data in a user-friendly way. TMCI is freely available at `https://github.com/schwartzlab-methods/too-many-cells-interactive`. An example tree from data within this paper is available at `https://tmci.schwartzlab.ca/`.

Key words: single-cell sequencing, data visualization, hierarchical clustering, big data, browser-based, interactive graphical user interface, drug-tolerant persister cells, cell line

Introduction

Single-cell sequencing quantifies transcriptomic and epigenomic activity at the resolution of individual cells, which enables unprecedented insight into the cellular landscape of biological processes and diseases. However, current approaches for single-cell visualization were not developed to scale with increasingly complex data produced by high-throughput sequencing technologies—both in terms of the number of measured cells and the number of features measured per cell.

A key component of single-cell analysis is to identify distinct cell states and types present within the experimental sample[1–4]. Most standard visualization workflows begin by collapsing the high-dimensional cell features (e.g. genes or chromosome regions) into two dimensions using techniques such as principal component analysis (PCA), t-distributed stochastic neighbor embedding (t-SNE) or uniform manifold approximation (UMAP)[5–7]. Current methods apply dimensionality reduction to make the data more amenable for analysis and visualization, a technique which often distorts distances between cells[8–12]. As a result, cells placed closer together on a scatter plot may not necessarily represent cells with higher biological similarity. Dimensionality reduction is commonly followed by unsupervised clustering algorithms such as k-means, Louvain, or Leiden, which are all limited to generating a single-resolution grouping that cannot simultaneously identify subpopulations and is heavily influenced by user-defined parameters[13]. By default, most analysis toolkits also apply clustering on low-dimensional embeddings to reduce computation time, thereby removing potential signals in the data for downstream interpretations. To overcome such limitations, we previously introduced TooManyCells—a suite of tools for cell-clade quantification[1,2]. The TooManyCells dendrogram depicts all cells starting at the root node, which become recursively bipartitioned at each subsequent child node based on similarity. While TooManyCells preserves distances between cells and presents multiple resolutions of cellular populations, the method produces static representations that generate complex trees with larger data sets.

In addition to methodological limitations of existing visualizations, many bioinformatic tools are out of reach because they require both computational expertise and biological insight for data exploration. To help bridge this gap, interactive tools such as CELLxGENE[14], Cirrocumulus[15], and others[16,17], facilitate visualization and inquiry of high-throughput single-cell data. More recent tools were designed to assist with specific challenges within analytical workflows such as read alignment[18], parameter selection[19], compute-intensive processes[20], cell type annotation[21], and lack of familiarity with programming[22,23]. However, their approaches towards single-cell data analysis remain fundamentally unchanged from that of conventional workflows and do not address scalability issues. Altogether, the limitations of current interactive visualization approaches inevitably affect our ability to interpret the underlying biology and represent a critical issue in high-throughput data analysis.

To address these limitations, we introduce TooManyCellsInteractive (TMCI), a browser-

based JavaScript application for interactive exploration of cell populations. TMCI is an easy-to-use tool that displays single-cell data as a radial tree of nested cell clusters and their relationships, and can be applied to a variety of different data types including gene expression from single-cell RNA sequencing (scRNA-seq)[1] and chromatin accessibility from single-cell assay for transposase-accessible chromatin (scATAC-seq)[2]. TMCI works seamlessly with TooManyCells dendrograms, so users can interactively explore the tree structure through a responsive dashboard to quickly and easily retrieve population statistics, manually or statistically alter cluster resolution of the tree, quickly overlay feature information, and batch export the display across thousands of trees (Figure 1). Here, we demonstrate TMCI's advantages over commonly-used visualization tools by benchmarking across several data sets and highlighting an example use case of TMCI to study drug-tolerance mechanisms across multiple cancer types. With an intuitive interface and flexible export system, TMCI is robust solution to visualize large single-cell data sets. TMCI is open source and packaged with all dependencies at `https://github.com/schwartzlab-methods/too-many-cells-interactive`. An example tree from data within this paper is available at `https://tmci.schwartzlab.ca/`.

Results

Implementation

TMCI consists of a browser-based graphical user interface (Figure 1), a web server, a relational database, a containerized runtime environment, and a collection of initialization and data processing scripts (Figure 2). The TMCI browser application is written in TypeScript, a statically-typed superset of JavaScript, and implements a variety of frameworks and libraries to provide a highly interactive graphical user interface. Principal UI elements include an interactive radial tree for data visualization and a dashboard-style panel of input controls enabling users to make real-time adjustments to their plots. Such adjustments include node filtering ("pruning"), scale modification, feature overlay, and manual position adjustment (Supplementary Note S1). For saving the tree, TMCI supports image exporting to both PNG and SVG formats.

The browser application's base architecture is provided by custom React.js components, while state management is handled by Redux and the interactive plots are created with D3.js, a widely-used low-level collection of data-visualization modules for scaling, event binding, DOM traversal, and high-performance animations (Figure 2).

The back-end Node application transpiles the Typescript to JavaScript using a Webpack bundler and serves it to the user's browser via an Express application (Figure 2). If users wish to include custom feature overlays in their plots, such as gene expression data, they may upload the data to the PostgreSQL database that has been configured to connect to the Node server (Supplementary Note S2).

Both the PostgreSQL database and the Node server run in Docker containers, for which TMCI provides a declarative configuration via Docker Compose. TMCI's containerized architecture allows it to be run on any computer with Docker installed, and the TMCI codebase includes Bash scripts intended as convenience wrappers around commonly-used Docker commands that can be easily extended for custom use.

Because D3.js has no strict browser dependencies, TMCI's radial tree plots can be rendered without a browser interface. TMCI provides both a Node script and a shell script to enable easy programmatic rendering. The scripts require an additional configuration JSON string that can be exported directly from the browser interface. Thus, users may refine their visualizations in the graphical environment and then re-use their configurations as templates for scripted batch processing on the server.

TMCI reduces time to display trees

To compare the computational time and memory of our TMCI approach to data visualization from both our original, static implementation as well as other commonly used single-cell data exploration tools CELLxGENE[14] and Cirrocumulus[15], we developed five benchmarks for common single-cell analyses: loading in all single-cell data and generating a visualization (*display with features*), overlaying colors on the visualization corresponding to a single feature annotation (*overlay feature*), batch processing five sequential feature overlays (*overlay multiple features*), adjusting the cluster resolution through tree pruning (i.e. reducing the size of the tree by collapsing child nodes into the parent node; *prune tree*), and rendering the visualization itself without loading the full read matrix (*tree display*). We ran these benchmarks using 54,220 cells from a scRNA-seq data set of 11 samples across five cancer cell lines (Figure 3a,b), 18,859 cells ("subset"; Figure 3c,d) and 41,668 cells (Figure 3e,f) from the Tabula Muris data set containing 10 mouse organs[24], and 483,152 cells from the Tabula Sapiens data set of 24 tissues and organs from the human body[25].

To assess a baseline performance of each program, we compared the time and memory needed to display trees without feature overlays on our cancer cell line data set, meaning that no matrix processing was required. Inputs for both programs were the tree and label files generated by TooManyCells and we ran each benchmark five times to account for potential variability. TMCI was 4 fold faster than TooManyCells in the cancer cell line data set (mean 1.07 s vs. 4.62 s, *t*-test: $p = 2.74 \times 10^{-18}$; Figure 3a) demonstrating an order of magnitude speed improvement with our new implementation. Importantly, this upgrade did not come at the cost of memory, as TMCI used $\sim$ 120 MB less memory than TooManyCells (mean 188 MB vs. 308 MB, *t*-test: $p = 1.87 \times 10^{-14}$; Figure 3b). As this benchmark did not alter the structure of the tree, we next compared tools by pruning the tree to have nodes containing no fewer than 1,000 cells. This additional processing resulted in TMCI using approximately the same amount of resources as the unpruned tree and TooManyCells increasing its performance to a mean of 1.64 s (*t*-test: $p = 2.39 \times 10^{-14}$) and 300 MB of RAM (*t*-test: $p = 5.56 \times 10^{-16}$; Figure 3a,b). While the performance increase of TMCI

over TooManyCells was consistent across data sets, some gains were 20 fold as with the larger Tabula Muris data set with insufficient memory to complete the visualization for TooManyCells on the Tabula Sapiens data set (Figure 3c-h, Supplementary Tables S1-S4).

Although TMCI outperformed with only tree display and processing, this benchmark did not account for matrix processing. As such, we next compared the performance of each program when rendering feature overlays, which introduces the resource-intensive task of retrieving expression data from the matrix. For a single feature on the cancer cell lines data set, TMCI outperformed TooManyCells in task duration (mean 554 s vs. 1,021 s, *t*-test: $p = 1.91 \times 10^{-15}$; Figure 3a). This advantage remained through TMCI's greatly reduced memory usage (mean 19.9 GB vs. 89.8 GB, *t*-test: $p = 2.11 \times 10^{-26}$; Figure 3b). While this test displayed significant memory gains for TMCI over TooManyCells, a more applicable benchmark is to batch process the creation of several graphics from a single tree with varying gene expression overlays. In this benchmark of five features, TMCI outperformed TooManyCells in both time (mean 577 s vs. 5,102 s, $p = 7.46 \times 10^{-18}$) and memory (mean 19.9 GB vs. 89.8 GB, $p = 2.44 \times 10^{-33}$; Figure 3a,b) usage due to the unique persistent feature database, which enables TMCI to generate any number of images after only a single data import operation. TooManyCells, on the other hand, must process the matrix for each new graphic, leading to a linear $\mathcal{O}(n)$ performance where n is the number of feature overlays requested. As a result, TMCI was able to generate ten trees with just 23 s longer than a single tree, while TooManyCells took ten times longer than a single tree. These observations were consistent through all data sets (Figure 3c-f, Supplementary Tables S1-S4).

To compare with non-tree based methods, we measured the performance of loading an entire single-cell data set and producing a visualization. For our cancer cell line data set, Cirrocumulus was the fastest (mean 331 s), with CELLxGENE (mean 528 s) and TMCI (mean 550 s) close behind, and TooManyCells being the slowest (mean 1,009 s; Figure 3a). Likewise, Cirrocumulus had the lowest memory usage (mean 9.34 GB), followed by CELLxGENE (mean 11.3 GB), TMCI (mean 19.9 GB), and TooManyCells (mean 89.8 GB; Figure 3b). Importantly, TMCI displays all cluster resolutions, while CELLxGENE and Cirrocumulus only show "flat" clusterings, even though TMCI has closer performance to these two tools than TooManyCells which is significantly more resource heavy (Supplementary Tables S1 and S2). These observations are consistent in the Tabula Muris data sets but not in the larger Tabula Sapiens, where TMCI has the second-lowest time and memory usage, outperforming CELLxGENE (Figure 3e-h, and Supplementary Tables S1-S4). Together, these benchmarks indicate not only the comparable performance of TMCI to a generate static and interactive tree of single-cell data compared to other tools across multiple clustering resolutions, but also its ability to quickly and efficiently batch process many trees at once.

Case study: TMCI effectively delineates subpopulations of cancer drug-tolerant persister cells

To demonstrate the utility of TMCI for quantification and visualization of relationships between diverse single-cell data sets, we explored the transcriptional differences induced by short- (2–3 days) and long-term (6–7 weeks) treatment of cancer cells *in vitro*. While treatment eliminates the majority of cancer cells, rare populations of drug-tolerant persister cells survive and may potentially act as a reservoir for drug-resistant growth[26]. Persister cells are characterized by a non-genetic, slow-cycling state that is reversible; upon drug holiday, persister cells are re-sensitized to treatment[27]. We sought to better understand the differences between short- and long-term treatment exposure in these persister cells using TMCI. To this end, we aggregated publicly available scRNA-seq data from five independent cancer persister-cell experiments across various disease areas and treatment modalities (Figure 4a and Table 1)[1,28–31]. The TMCI visualization identified distinct separation between cancer cell lines, followed by division of control and treatment arms (Figure 4b). This hierarchy suggests that cells of a given cancer type, regardless of drug treatment, are more transcriptionally similar to one another than persister cells across cancer types for most populations.

TMCI identified differentially expressed *ID2* across persister-cell populations

In order to understand how survival programs could be affected by the duration of treatment, we sought to characterize the unique expression profiles among persister-cell populations. We identified differentially expressed genes between control and persister cells of each cell line separately and aggregated the complete list of genes using rank product analysis[32]. The batch functionality of TMCI allowed us to efficiently visualize the distribution of top-ranking most differentially expressed genes across the entire data-set collection. From these visualizations, we identified *ID2* as one of the most highly upregulated genes across long-term treated cells in comparison to controls (rank product: 4, permutation test: $p < 2.22 \times 10^{-16}$), but not among the short-term treated cells (rank product: 380, permutation test: $p < 2.22 \times 10^{-16}$) (Figure 4c and Supplementary Tables S5 and S6). Comparison of *ID2* expression between each control and corresponding treatment arm showed a significant increase of $\log_2$ fold change values for all cell lines (Mann-Whitney U test: $p < 0.05$), regardless of treatment duration, with the exception of short-term treated DND-41 cells (Figure 4d and Supplementary Table S7). *ID2* is known to play a role in tumorigenesis as a key regulator of cell-cycle progression and overexpression of *ID2* in cell-line experiments modulates proliferative capacity and cell invasiveness[33,34]. Differential *ID2* expression in our analysis suggests varying proliferative activity between treatment durations.

From the tree structure, we noticed a subset of treated MDA-MB-231 breast cancer cells with particularly high *ID2* expression that did not group together with the predominant cell line cluster (Supplementary Figure S1a). Rather, this subset in node 4 grouped more closely

with PC9 lung cancer cells than with other cells of the same disease type and treatment condition in node 126. To explore the differences underlying this distinct cell state, we performed differential expression analysis comparing treated MDA-MB-231 cells of node 4 against node 126 (Supplementary Table S8). Metascape analysis of the top 100 most downregulated genes identified "negative regulation of cell differentiation" (hypergeometric test: $p = 4.68 \times 10^{-4}$) and "PTEN regulation" (hypergeometric test: $p = 9.55 \times 10^{-3}$) as significantly enriched pathways (Supplementary Figure S1b). We corroborated these results through gene set enrichment analysis, which identified signals of dysregulated *ID2*, *KRAS*, *PTEN*, and *YAP1* expression among the most differentially represented oncogenic signatures (Supplementary Table S9). Interestingly, many of these signatures were derived from RNA interference screens of *KRAS*G13D-mutant cell lines for synthetic lethal targets[35]. MDA-MB-231 cells also harbor this oncogenic mutation, which drives constitutive signaling of the $KRAS^{G13D}$ protein[36,37]. However, we found significant downregulation of *KRAS* expression and under-representation of its target genes, accompanied by upregulation of *KRAS*-mutant synthetic lethal vulnerabilities *TBK1*, *YAP1* and *STK33* within the subset of interest[38,39] (Mann-Whitney U tests: *KRAS* $\log_2$FC −1.80, $p = 1.41 \times 10^{-36}$; *TBK1* $\log_2$FC 0.682, $p = 0.0320$; *YAP1* $\log_2$FC 0.932, $p = 1.21 \times 10^{-7}$; *STK33* $\log_2$FC 1.312, $p = 0.700$; Supplementary Table S8 and Supplementary Figure S1c). Altogether, these findings suggest multiple treated populations, one of which undergoes activation of *KRAS*-mutant compensatory signaling within a subset of treated MDA-MB-231 cells and demonstrate some of the advantages of a tree-based approach for single cell analysis.

TMCI identifies distinct proliferation mechanisms within persister-cell populations

To interrogate the ongoing biological mechanisms within short and long-term treated persister-cell populations, we performed pathway analysis using the top 100 upregulated differentially-expressed genes in the treated cells. Metascape[40] analysis of the differentially-expressed genes from short-term treated cells identified "negative regulation of cell population proliferation" as a key biological process (hypergeometric test: $p = 2.88 \times 10^{-4}$; Figure 4e). Conversely, the same analysis performed on differentially-expressed genes identified "cell population proliferation" enrichment in long-term treated cells, suggesting an increase of cellular proliferation across pathways (hypergeometric test: $p = 4.89 \times 10^{-4}$; Figure 4e). Subsequent exploration of the full list of differentially expressed genes using Gene Set Enrichment Analysis[41] returned markedly distinct biological programs between the short and long-term treated populations. Among short-term treated populations, the most significantly decreased hits were found to be associated with various proliferation and cell-cycle-regulation programs. In line with our previous findings, these programs were not significantly downregulated among long-term treated cells (Supplementary Table S10).

Among these gene sets, we found the expression of "FISCHER_G2_M_CELL_CYCLE" significantly decreased among short-term treated cells (NES $= -2.30$, Kolmogorov-Smirnov test: $p < 2.22 \times 10^{-16}$) but not among long-term treated cells (NES $= -0.891$, Kolmogorov-Smirnov test: $p = 0.747$; Figure 4f-i and Supplementary Figure S2a). Consistent with this observation, additional G2M checkpoint and E2F target gene sets showed similar patterns (Supplementary Table S11). These findings suggest that persister cells utilize distinct pathways associated with modulation of proliferation and cell cycling throughout the duration of treatment.

TMCI identifies subpopulations with highly expressed diapause programs

As we identified proliferation and cell-cycle factors associated with treatment duration, we were interested in understanding the temporal expression of diapause programs within the various persister-cell populations. Diapause is a reversible state of suspended embryonic development triggered by adverse environmental conditions[42]. Similarly, persister cells which survive throughout exposure to treatment undergo transcriptional adaptations resembling a diapause-like state[43,44]. Overlaying diapause gene signature scores on the tree structure showed enrichment in all treated subpopulations compared to controls (Figure 4j and Supplementary Figure S2b).

Comparison between each control and treatment arm showed significantly increased diapause signature scores in all treated cell lines, again regardless of treatment duration (Mann-Whitney U test: $p < 0.05$; Figure 4k, Supplementary Figure S2c-f, and Supplementary Table S12). For DND-41, which includes measurements of both short- and long-term treatment durations, the median diapause signature score increased from control to short-term to long-term, suggesting a direct correlation between diapause gene signature scores and treatment duration. Confirming that the easily-seen difference in diapause signature scores within each cell line was significant, we compared the TMCI visualization against a traditional scatter plot generated with CELLxGENE (Supplementary Figure S2g-h, Supplementary Figure S3, and Supplementary Note S4). Although TMCI and CELLxGENE had the same diapause signature scores, the significantly different subpopulations were more easily seen in TMCI's tree. Together, our analysis points to persister cells with different proliferation activity depending on treatment duration.

Discussion

As high-throughput single-cell technologies continue to measure increasing numbers of cells, we need new visualization tools to better identify and interpret cell states. Here we present TMCI as a powerful, interactive solution that simplifies data exploration of large data sets. These visualizations are intuitive, supporting easy tree manipulation through statistical or manual pruning, color mapping, feature overlays, and more. With these features, identification of rare cellular populations is straightforward compared to

previous iterations of single-cell data figures. Importantly, these benefits are not at the cost of performance, with TMCI either outperforming or on-par with alternative interactive visualizations. As we implemented TMCI as a web server, users can easily and quickly access large data sets with little computational impact on their local host. As a result of TMCI's speed, its batch processing capability allows for quick plotting of thousands of trees derived from a single, manually-customized tree.

Using the numerous features afforded by TMCI, we delineated cellular populations from drug-treated cancer cell lines and identified distinct transcriptional programs between short- and long-term treated cell lines. These programs included cell-proliferation pathways downregulated in short-term persister cell states which are then subsequently lost in the long-term cellular populations across all cancer types measured. This finding extended to the diapause signature, which was increased in persister cells, in concordance with previous studies, but here across cancer type. Together, TMCI identified transcriptional programs that are dependent on treatment duration, suggesting further investigation on the timing of treatment for persister cells.

Although TMCI is a feature-rich application for tree structure exploration, there are several future directions that could enhance TMCI's capabilities. While projection-based visualizations such as t-SNE and UMAP have limited capabilities in identifying cell relationships, they are still widely used among the single-cell sequencing analysis community. To link these visualizations together, a new user interface could be created for simultaneous investigation similar to Sleepwalk[9]. These combinations of multiple embeddings may also include other dashboard features such as gene expression heatmaps and enriched pathways. Furthermore, TMCI currently displays relationships generated from a single data modality. As new multi-omic technologies sequence both RNA and chromatin accessibility or protein from the same cell, there exists new opportunities for TMCI to integrate multiple data modalities in a tree structure. In the meantime, through our application to drug treated cancer cell lines, we show that big data visualization tools will be necessary as available data grows, and we provide TMCI as a solution for visualizing tree-based relationships in such data.

Materials and Methods

Benchmarks

We performed benchmarks using an AWS EC2 instance running Ubuntu 20.04 and Docker 20.10.17 with 64x Intel Xeon Platinum 8375C CPU @ 2.90 GHz and 534 GB RAM. We compared CELLxGENE[14], Cirrocumulus[15], TooManyCells[1,2], and TMCI using 54,220 cells from five cancer cell lines, 41,668 cells from the Tabula Muris data set[24], a smaller subset of 18,859 cells from the Tabula Muris data set, and 483,152 cells from the Tabula Sapiens

data set[25].

For each method, we devised five benchmarks for compute time and memory usage, some of which were unique to tree-based approaches. For all methods, we loaded all single-cell data and ran the default options to generate visualizations (*display with features*). Based on this benchmark, we also overlaid a single color on the visualization corresponding to a single feature annotation (*overlay feature*) or also used batch processing for five sequential feature overlays (*overlay multiple features*). Specific to tree-based approaches, we measured tree pruning by collapsing child nodes into the parent node (*prune tree*). We also benchmarked performance when only displaying the full tree visualization without loading the entire single-cell matrix (*tree display*). We ran each benchmark five times to account for variability in processing time and memory.

Preprocessing of drug-treated cancer scRNA-seq data

To demonstrate the utility of TMCI, we investigated drug-tolerant persister-cell populations which are capable of surviving anti-cancer drug treatment through non-genetic programming of reversible mechanisms[27]. We aggregated publicly available scRNA-seq data from five *in vitro* persister experiments including prostate cancer, line melanoma, non-small cell lung cancer, breast cancer, and T-cell acute lymphoblastic leukemia cell lines (Table 1). The duration of anti-cancer drug treatment for each cell lines varied from short-term (2–3 days) to long-term (6–7 weeks), enabling the identification of persister cells across cancer types and time. All datasets were previously generated using similar library preparation methods (10x Genomics 3’ Single Cell Gene Expression), sequencing platforms (Illumina), and alignment pipelines (Cell Ranger). After manual checks to verify that the files contained raw read count data, we aggregated the matrices using AnnData and Scanpy[4] tools in Python. We applied all normalization and filtering using the TooManyCells command-line tool, based on its original default parameters of term frequency-inverse document frequency (TF-IDF) normalization and filtering for cells expressing at least 250 transcripts and genes detected in at least one cell[1]. For other batch-effect correction techniques, we recommend using our TooManyCells (à la Python) Python implementation which better handles non-count, transformed embeddings (Supplementary Note S3).

Generating drug-treated cancer cell trees

After data normalization and filtering, we used TooManyCells to generate a tree and identify transcriptionally distinct subpopulations within our data set[1]. In brief, TooManyCells implements a matrix-free hierarchical spectral clustering approach[45] to recursively partition scRNA-seq cell data into similar groups, and uses Newman-Girvan modularity[46] as an indicator for reaching a leaf in the tree. The resulting tree structure depicts all cells at the

central root node, with subdividing branches for each group partition until any additional split would be considered random. This information is encoded in the `cluster_tree.json` output file and can be viewed interactively through TMCI. We used the resulting tree structure groupings as input for TMCI, through which we applied minimum distance search pruning at a cutoff of 0.019 to improve the visibility of small sub-populations.

Measuring differential expression across cellular populations

Using the tree structure, we conducted differential gene expression analysis between control and persister cell states of each cell individually, using the TooManyCells "differential" functionality with upper quartile normalized read counts. From the resulting $\log_2$ fold change values, we aggregated a list of differentially expressed genes across cell lines using rank product analysis[32]. These results identified genes which are more broadly associated with the persister state across drug treatments and cancer disease types. We used the batch functionality of TMCI to iterate through the list of top-ranking gene targets and visually identified *ID2* as a potential target of interest on account of its high expression among long-term treated cell lines, which we did not observe across short-term treated persister cells. These findings corroborated with statistical comparisons of expression between treatment conditions within each given cell line, highlighting *ID2* as a target of interest.

To explore the biological mechanisms associated with each cell state, we conducted gene set enrichment analysis[41] across the tree structure. For this analysis, we calculated the $\log_2$ fold change values of each node against all other cells using the methods from Scanpy "rank_genes_groups". With each ordered gene list, we ran the GSEApy "preranked" module with MSigDB Hallmark, C2 (curated) and C6 (oncogenic) gene sets[47]. We used two-sided statistical tests for all analyses.

Abbreviations

GSEA: Gene Set Enrichment Analysis

NES: Normalized Enrichment Score

PCA: Principal Component Analysis

RAM: Random-Access Memory

TMC: TooManyCells

TMCI: TooManyCellsInteractive

UMAP: Uniform Manifold Approximation and Projection

scATAC-seq: Single-cell Assay for Transposase-Accessible Chromatin

scRNA-seq: Single-cell RNA sequencing

t-SNE: t-distributed Stochastic Neighbor Embedding

Availability of Supporting Source Code and Requirements

1. **Project name:** TooManyCellsInteractive
   Project home page: `https://github.com/schwartzlab-methods/too-many-cells-interactive`
   Operating system: Platform independent
   Programming language: TypeScript, JavaScript
   Other requirements: Docker, Docker-compose
   License: GNU General Public License v3.0
   RRID: SCR_025315

2. **Project name:** TooManyCells (à la Python)
   Project home page: `https://github.com/schwartzlab-methods/too-many-cells-python`
   Operating system: Platform independent
   Programming language: Python
   Other requirements: Graphviz (`https://graphviz.org/`)
   License: GNU Affero General Public License v3.0
   PyPi: toomanycells (`https://pypi.org/project/toomanycells/`)
   RRID: SCR_025327

TMCI is available at `https://github.com/schwartzlab-methods/too-many-cells-interactive` or on figshare at `https://figshare.com/articles/software/too-many-cells-interactive-main_zip/24247426`, with a tutorial at `https://schwartzlab-methods.github.io/too-many-cells-interactive/`. TMCI will read any tree in the appropriate format, including trees generated by the original TooManyCells command-line tool located at `https://github.com/GregorySchwartz/too-many-cells`. We also introduce TooManyCells (à la Python), a new Python implementation of the tree creation algorithm that includes functions for rendering tree objects with TMCI. TooManyCells (à la Python) is located at `https://github.com/schwartzlab-methods/too-many-cells-python` and is fully compatible with Scanpy[4]. Code for analyses within this paper are available at `https://github.com/schwartzlab-methods/too-many-cells-interactive-paper-analyses`.

Authors Contributions

G. W. S. conceived and supervised the project. C. K. developed the tool and benchmarks. C. K. ran and analyzed benchmarks. C. J. L. collected, ran, and analyzed cancer cell line data. C. K., C. J. L., and G. W. S. wrote the manuscript.

Funding

This work was supported by the University of Toronto Data Sciences Institute Research Software Development Support Program (G. W. S.), the Canadian Cancer Society Challenge Grant (grant 707484; G. W. S.), the Natural Sciences and Engineering Research Council of Canada (grants RGPIN-2023-04713 and DGECR-2023-00395; G. W. S.), the Social Sciences and Humanities Research Council (grant NFRFE-2022-00681; G. W. S.), the Canada Research Chairs Program (G. W. S.), the Princess Margaret Cancer Foundation (G. W. S.), and the University of Toronto Data Sciences Institute Doctoral Student Fellowship (C. J. L.).

Data Availability

The GEO accession numbers for each data set reported in this paper are GSM5155455 and GSM5155456 (prostate cancer); GSM4932163 and GSM4932166 (melanoma); GSM3972651 and GSM3972652 (non-small cell lung cancer); GSM4684556 and GSM4684557 (breast cancer); GSM4121361, GSM4121362, and GSM4121364 (T-cell acute lymphoblastic leukemia).

Competing Interests

The authors declare no competing interests.

References

1. Schwartz, G. W. *et al.* TooManyCells Identifies and Visualizes Relationships of Single-Cell Clades. *Nat. Methods* **17,** 405–413 (2020).
2. Schwartz, G. W., Zhou, Y., Petrovic, J., Pear, W. S. & Faryabi, R. B. TooManyPeaks Identifies Drug-Resistant-Specific Regulatory Elements from Single-Cell Leukemic Epigenomes. *Cell Rep.* **36** (2021).
3. Satija, R., Farrell, J. A., Gennert, D., Schier, A. F. & Regev, A. Spatial Reconstruction of Single-Cell Gene Expression Data. *Nat. Biotechnol.* **33,** 495–502 (2015).
4. Wolf, F. A., Angerer, P. & Theis, F. J. SCANPY: Large-Scale Single-Cell Gene Expression Data Analysis. *Genome Biol.* **19,** 15 (2018).
5. McInnes, L., Healy, J. & Melville, J. UMAP: Uniform Manifold Approximation and Projection for Dimension Reduction. *ArXiv180203426 Cs Stat* (2018).
6. Van der Maaten, L. & Hinton, G. Visualizing Data Using T-SNE. *J. Mach. Learn. Res.* **9,** 2579–2605 (2008).
7. Xiang, R. *et al.* A Comparison for Dimensionality Reduction Methods of Single-Cell RNA-seq Data. *Front. Genet.* **12** (2021).
8. Wattenberg, M., Viégas, F. & Johnson, I. How to Use T-SNE Effectively. *Distill* **1,** e2 (2016).
9. Ovchinnikova, S. & Anders, S. Exploring Dimension-Reduced Embeddings with Sleepwalk. *Genome Res.* **30,** 749–756 (2020).
10. Cooley, S. M., Hamilton, T., Aragones, S. D., Ray, J. C. J. & Deeds, E. J. *A Novel Metric Reveals Previously Unrecognized Distortion in Dimensionality Reduction of scRNA-seq Data* 2022.
11. Chari, T., Banerjee, J. & Pachter, L. *The Specious Art of Single-Cell Genomics* 2021.
12. Kobak, D. & Berens, P. The Art of Using T-SNE for Single-Cell Transcriptomics. *Nat. Commun.* **10,** 1–14 (2019).
13. Traag, V. A., Waltman, L. & van Eck, N. J. From Louvain to Leiden: Guaranteeing Well-Connected Communities. *Sci. Rep.* **9,** 5233 (2019).
14. Megill, C. *et al.* Cellxgene: a performant, scalable exploration platform for high dimensional sparse matrices. *bioRxiv,* 2021–04 (2021).
15. Li, B. *et al.* Cumulus Provides Cloud-Based Data Analysis for Large-Scale Single-Cell and Single-Nucleus RNA-seq. *Nat. Methods* **17,** 793–798 (2020).
16. Zheng, G. X. Y. *et al.* Massively Parallel Digital Transcriptional Profiling of Single Cells. *Nat. Commun.* **8,** 14049 (2017).

17. Speir, M. L. *et al.* UCSC Cell Browser: visualize your single-cell data. *Bioinformatics* **37,** 4578–4580 (2021).

18. Prieto, C., Barrios, D. & Villaverde, A. SingleCAnalyzer: Interactive Analysis of Single Cell RNA-Seq Data on the Cloud. *Front. Bioinform.* **2** (2022).

19. Innes, B. T. & Bader, G. D. scClustViz – Single-cell RNAseq cluster assessment and visualization. *F1000Research* **7,** ISCB Comm J–1522 (2019).

20. Tabaka, M., Gould, J. & Regev, A. *scSVA: an interactive tool for big data visualization and exploration in single-cell omics* 2019.

21. Hasanaj, E., Wang, J., Sarathi, A., Ding, J. & Bar-Joseph, Z. Interactive single-cell data analysis using Cellar. *Nat. Commun.* **13,** 1998 (2022).

22. Hillje, R., Pelicci, P. G. & Luzi, L. Cerebro: interactive visualization of scRNA-seq data. *Bioinformatics* **36,** 2311–2313 (2020).

23. Kotliar, D. & Colubri, A. Sciviewer enables interactive visual interrogation of single-cell RNA-Seq data from the Python programming environment. *Bioinformatics* **37,** 3961–3963 (2021).

24. The Tabula Muris Consortium *et al.* Single-Cell Transcriptomics of 20 Mouse Organs Creates a Tabula Muris. *Nature* **562,** 367–372 (2018).

25. CONSORTIUM, T. T. S. The Tabula Sapiens: A multiple-organ, single-cell transcriptomic atlas of humans. *Science* **376,** eabl4896 (2022).

26. Glickman, M. S. & Sawyers, C. L. Converting Cancer Therapies into Cures: Lessons from Infectious Diseases. *Cell* **148,** 1089–1098 (2012).

27. Sharma, S. V. *et al.* A Chromatin-Mediated Reversible Drug-Tolerant State in Cancer Cell Subpopulations. *Cell* **141,** 69–80 (2010).

28. Taavitsainen, S. *et al.* Single-cell ATAC and RNA sequencing reveal pre-existing and persistent cells associated with prostate cancer relapse. *Nat. Commun.* **12,** 5307 (2021).

29. Celeste, F. & Powers, S. *Induction of Multiple Alternative Mitogenic Signaling Pathways Accompanies Emergence of Slowly Growing Drug-Tolerant Cancer Cell* (2022).

30. Aissa, A. F. *et al.* Single-cell transcriptional changes associated with drug tolerance and response to combination therapies in cancer. *Nat. Commun.* **12,** 1628 (2021).

31. Johnson, K. E. *et al.* Integrating transcriptomics and bulk time course data into a mathematical framework to describe and predict therapeutic resistance in cancer. *Phys. Biol.* **18,** 016001 (2020).

32. Breitling, R., Armengaud, P., Amtmann, A. & Herzyk, P. Rank Products: A Simple, yet Powerful, New Method to Detect Differentially Regulated Genes in Replicated Microarray Experiments. *FEBS Letters* **573,** 83–92 (2004).

33. Itahana, Y. *et al.* Role of Id-2 in the Maintenance of a Differentiated and Noninvasive Phenotype in Breast Cancer Cells1. *Cancer Res.* **63,** 7098–7105 (2003).

34. Stighall, M., Manetopoulos, C., Axelson, H. & Landberg, G. High ID2 protein expression correlates with a favourable prognosis in patients with primary breast cancer and reduces cellular invasiveness of breast cancer cells. *Int. J. Cancer* **115,** 403–411 (2005).

35. Barbie, D. A. *et al.* Systematic RNA Interference Reveals That Oncogenic KRAS-driven Cancers Require TBK1. *Nature* **462,** 108–112 (2009).

36. Kozma, S. C. *et al.* The Human C-Kirsten *Ras* Gene Is Activated by a Novel Mutation in Codon 13 in the Breast Carcinoma Cell Line MDA-MB231. *Nucleic Acids Research* **15,** 5963–5971 (1987).

37. Karapetis, C. S. *et al. K-Ras* Mutations and Benefit from Cetuximab in Advanced Colorectal Cancer. *New England Journal of Medicine* **359,** 1757–1765 (2008).

38. Aguirre, A. J. & Hahn, W. C. Synthetic Lethal Vulnerabilities in *KRAS* -Mutant Cancers. *Cold Spring Harbor Perspectives in Medicine* **8,** a031518 (2018).

39. Scholl, C. *et al.* Synthetic Lethal Interaction between Oncogenic KRAS Dependency and STK33 Suppression in Human Cancer Cells. *Cell* **137,** 821–834 (2009).

40. Zhou, Y. *et al.* Metascape Provides a Biologist-Oriented Resource for the Analysis of Systems-Level Datasets. *Nat. Commun.* **10,** 1523 (2019).

41. Subramanian, A. *et al.* Gene Set Enrichment Analysis: A Knowledge-Based Approach for Interpreting Genome-Wide Expression Profiles. *Proc. Natl. Acad. Sci.* **102,** 15545–15550 (2005).

42. Fenelon, J. C. & Renfree, M. B. The history of the discovery of embryonic diapause in mammals. *Biol. Reprod.* **99,** 242–251 (2018).

43. Rehman, S. K. *et al.* Colorectal Cancer Cells Enter a Diapause-like DTP State to Survive Chemotherapy. *Cell* **184,** 226–242.e21 (2021).

44. Dhimolea, E. *et al.* An Embryonic Diapause-like Adaptation with Suppressed Myc Activity Enables Tumor Treatment Persistence. *Cancer Cell* **39,** 240–256.e11 (2021).

45. Shu, L., Chen, A., Xiong, M. & Meng, W. *Efficient SPectrAl Neighborhood Blocking for Entity Resolution* in (IEEE, 2011), 1067–1078.

46. Newman, M. E. J. & Girvan, M. Finding and Evaluating Community Structure in Networks. *Phys. Rev. E* **69** (2004).

47. Liberzon, A. *et al.* Molecular signatures database (MSigDB) 3.0. *Bioinformatics* **27,** 1739–1740 (2011).

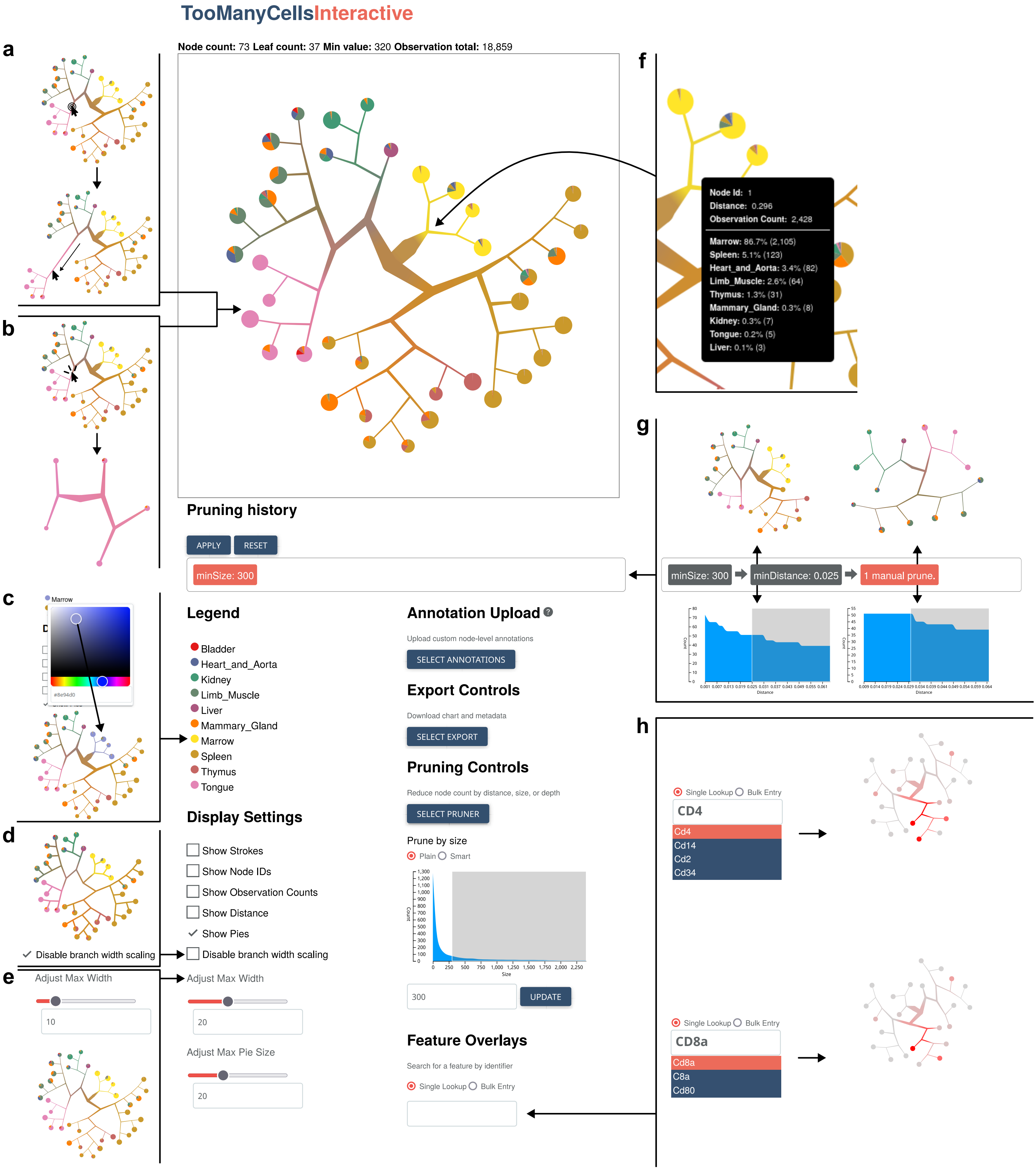
TooManyCellsInteractive
a
b
c
d
e
f
g
h
Node count: 73 Leaf count: 37 Min value: 320 Observation total: 18,859
Node Id: 1
Distance: 0.296
Observation Count: 2,428
Marrow: 86.7% (2,105)
Spleen: 5.1% (123)
Heart_and_Aorta: 3.4% (82)
Limb_Muscle: 2.6% (64)
Thymus: 1.3% (31)
Mammary_Gland: 0.3% (8)
Kidney: 0.3% (7)
Tongue: 0.2% (5)
Liver: 0.1% (3)
Pruning history
APPLY
RESET
minSize: 300
minSize: 300
minDistance: 0.025
1 manual prune.
Legend
Bladder
Heart_and_Aorta
Kidney
Limb_Muscle
Liver
Mammary_Gland
Marrow
Spleen
Thymus
Tongue
Marrow
#8e94d0
Display Settings
Show Strokes
Show Node IDs
Show Observation Counts
Show Distance
Show Pies
Disable branch width scaling
Disable branch width scaling
Adjust Max Width
10
Adjust Max Width
20
Adjust Max Pie Size
20
Annotation Upload
Upload custom node-level annotations
SELECT ANNOTATIONS
Export Controls
Download chart and metadata
SELECT EXPORT
Pruning Controls
Reduce node count by distance, size, or depth
SELECT PRUNER
Prune by size
Plain
Smart
Count
Size
300
UPDATE
Feature Overlays
Search for a feature by identifier
Single Lookup
Bulk Entry
Distance
Single Lookup
Bulk Entry
CD4
Cd4
Cd14
Cd2
Cd34
Single Lookup
Bulk Entry
CD8a
Cd8a
C8a
Cd80

Figure 1: Overview of the TMCI output interface. **a,b** Direct interactions with the main interface. The user may manually edit the tree in the main interface by stretching or shrinking branches (**a**) or selecting a new tree root (**b**). **c** Color picker for cell labels through hex value or slider when selecting the label of choice in the legend. **d,e** Visualization features of the tree branches and nodes, including the disabling of branch scaling (**d**) and adjusting the width of branches (**e**), among other visualization features. **f** Live-updating tooltips containing statistics for each node. **g** Breadcrumb toolbar containing previous structural changes as the user interactively prunes the tree based on the distribution of nodes. **h** Fuzzy-search bar to see the overlay of a feature on each node in the tree, such as gene expression for each cellular population. The user may select one or several features through the fuzzy-search bar and select thresholds for "high" and "low" cutoffs for simultaneous feature overlays (e.g. both *CD4* and *CD8a*).

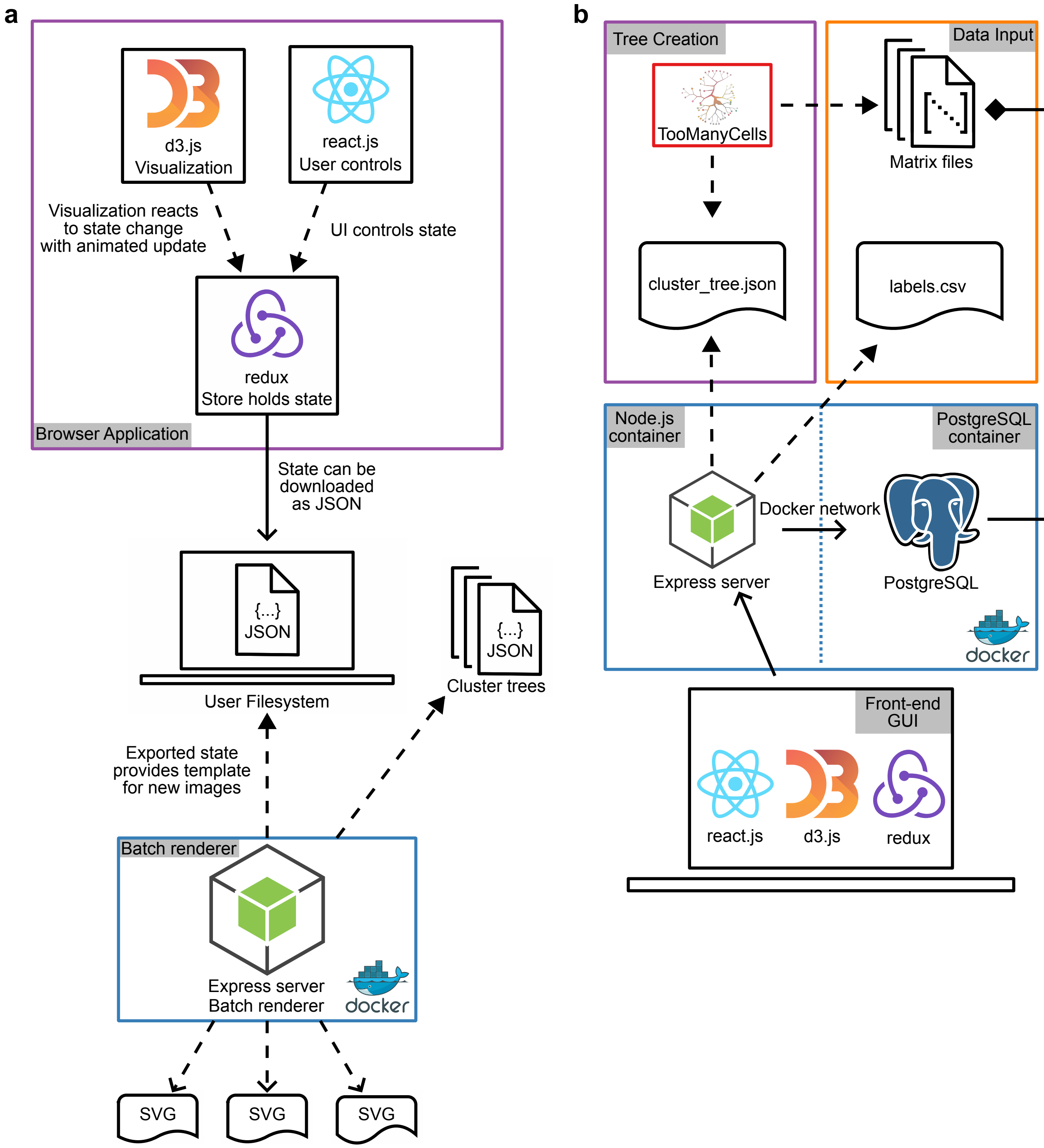
a
d3.js
Visualization
react.js
User controls
Visualization reacts
to state change
with animated update
UI controls state
redux
Store holds state
Browser Application
State can be
downloaded
as JSON
{...}
JSON
User Filesystem
{...}
JSON
Cluster trees
Exported state
provides template
for new images
Batch renderer
Express server
Batch renderer
docker
SVG
SVG
SVG
b
Tree Creation
TooManyCells
cluster_tree.json
Data Input
Matrix files
labels.csv
Node.js
container
PostgreSQL
container
Docker network
Express server
PostgreSQL
docker
Front-end
GUI
react.js
d3.js
redux

Figure 2: The architecture for TMCI. **a** The front-end architecture of TMCI. The user interacts with the D3 tree visualization and React user interface which sends state-change requests to Redux. This state-tracking feature enables batch processing: the user may upload a configuration state which the Express server will read without loading the graphical user interface and automatically export the corresponding SVG. **b** The back-end Express server container takes as input the tree structure and cell label files in the Node application. Similarly, a PostgreSQL container reads the matrix files containing the count matrices with features such as gene expression or chromatin accessibility. The Express container manages feature overlays on the tree through PostgreSQL queries in response to front-end requests. Flow charts are Unified Modeling Language structured diagrams where dashed closed arrows indicate dependencies, dashed open arrows indicate artifacts, solid closed arrows indicate relationships, and solid diamonds are compositions.

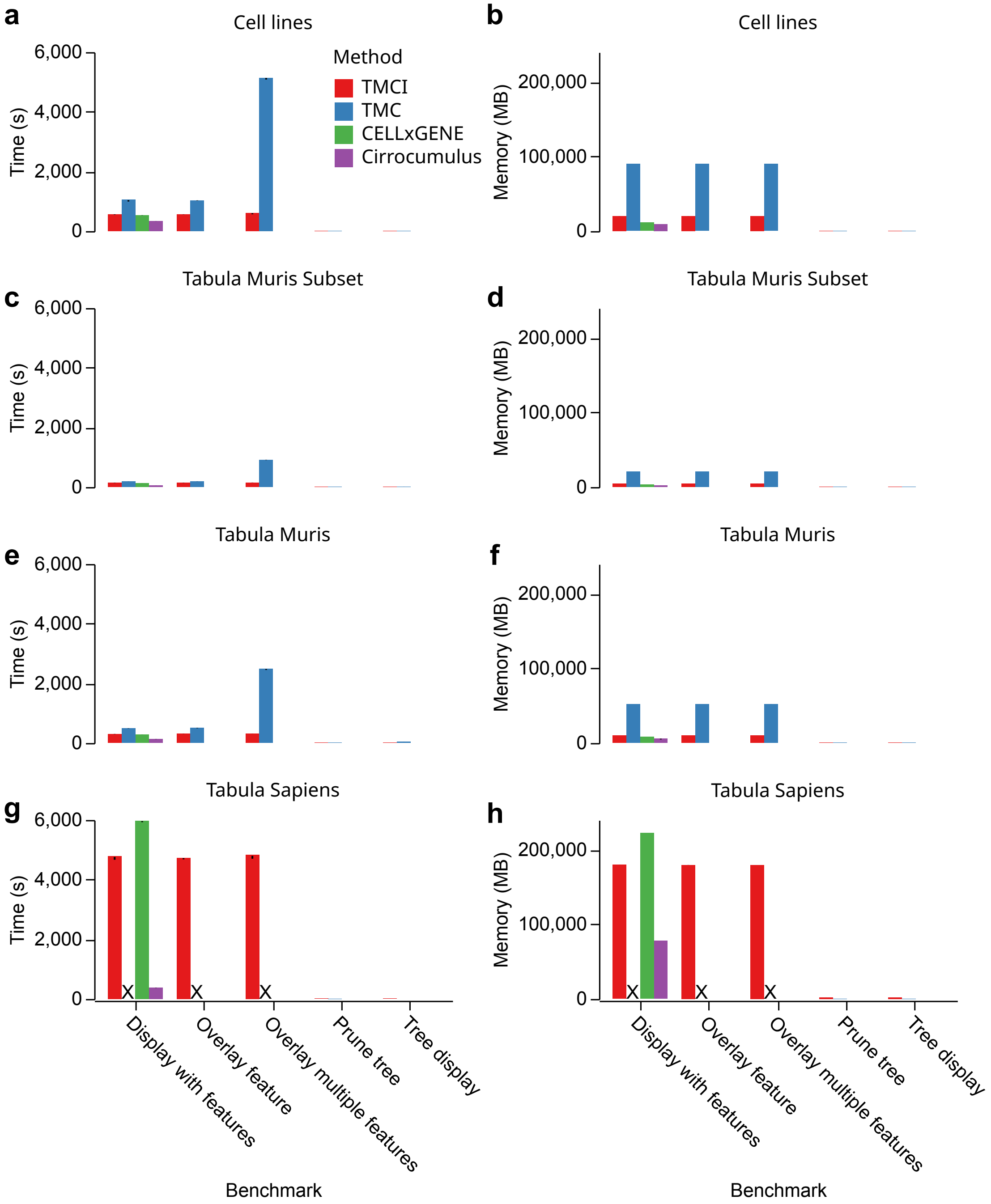
a
Cell lines
Method
TMCI
TMC
CELLxGENE
Cirrocumulus
Time (s)
6,000
4,000
2,000
0
b
Cell lines
Memory (MB)
200,000
100,000
0
c
Tabula Muris Subset
d
Tabula Muris Subset
e
Tabula Muris
f
Tabula Muris
g
Tabula Sapiens
h
Tabula Sapiens
X
Display with features
Overlay feature
Overlay multiple features
Prune tree
Tree display
Benchmark

Figure 3: Comparative analysis of performance. **a-f** Comparisons on the *x*-axis including, from left to right, loading a count matrix and displaying a visualization (all programs), overlaying a single feature on a tree (tree programs only), batch processing five features on a tree (tree programs only), pruning a larger tree (tree programs only), and rendering a tree without the count matrix loaded (tree programs only). Comparisons were split by 11 samples from five cancer cell lines in response to drug treatment (**a,b**), a set of 10 samples from mouse tissues[24] (**c,d**), or a set 24 samples from mouse tissues (**e,f**), and 24 human tissues and organs[25], measuring time (**a,c,e,g**) or memory usage (**b,d,f,h**). TMC: TooManyCells, X: Incomplete due to insufficient memory.

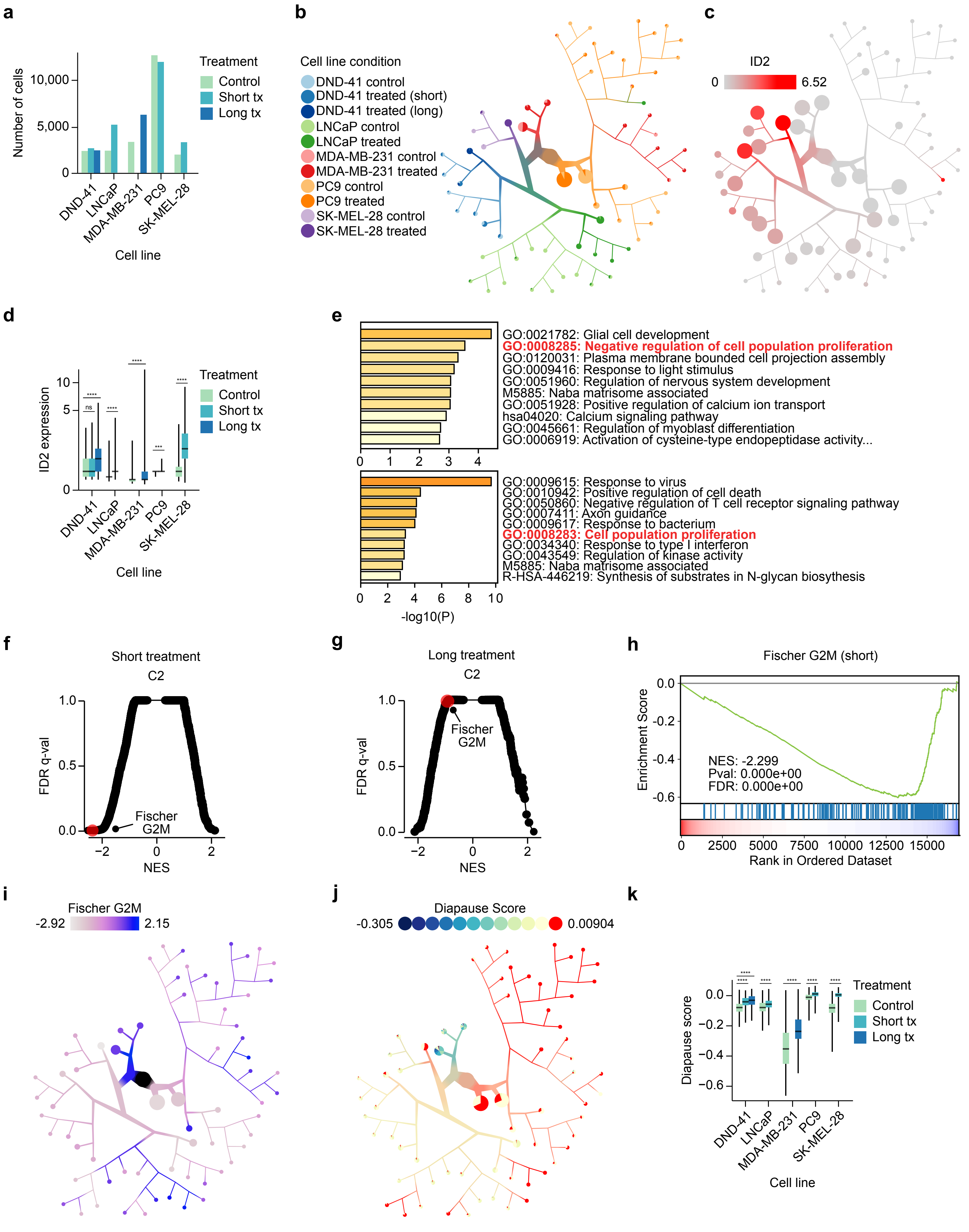
a
Number of cells
10,000
5,000
0
Treatment
Control
Short tx
Long tx
DND-41
LNCaP
MDA-MB-231
PC9
SK-MEL-28
Cell line
b
Cell line condition
DND-41 control
DND-41 treated (short)
DND-41 treated (long)
LNCaP control
LNCaP treated
MDA-MB-231 control
MDA-MB-231 treated
PC9 control
PC9 treated
SK-MEL-28 control
SK-MEL-28 treated
c
ID2
0
6.52
d
ID2 expression
10
5
0
ns
Treatment
Control
Short tx
Long tx
DND-41
LNCaP
MDA-MB-231
PC9
SK-MEL-28
Cell line
e
GO:0021782: Glial cell development
GO:0008285: Negative regulation of cell population proliferation
GO:0120031: Plasma membrane bounded cell projection assembly
GO:0009416: Response to light stimulus
GO:0051960: Regulation of nervous system development
M5885: Naba matrisome associated
GO:0051928: Positive regulation of calcium ion transport
hsa04020: Calcium signaling pathway
GO:0045661: Regulation of myoblast differentiation
GO:0006919: Activation of cysteine-type endopeptidase activity...
0 1 2 3 4
GO:0009615: Response to virus
GO:0010942: Positive regulation of cell death
GO:0050860: Negative regulation of T cell receptor signaling pathway
GO:0007411: Axon guidance
GO:0009617: Response to bacterium
GO:0008283: Cell population proliferation
GO:0034340: Response to type I interferon
GO:0043549: Regulation of kinase activity
M5885: Naba matrisome associated
R-HSA-446219: Synthesis of substrates in N-glycan biosynthesis
0 2 4 6 8 10
-log10(P)
f
Short treatment
C2
FDR q-val
1.0
0.5
0.0
Fischer
G2M
−2
0
2
NES
g
Long treatment
C2
FDR q-val
1.0
0.5
0.0
Fischer
G2M
−2
0
2
NES
h
Fischer G2M (short)
Enrichment Score
0.0
-0.2
-0.4
-0.6
NES: -2.299
Pval: 0.000e+00
FDR: 0.000e+00
0 2500 5000 7500 10000 12500 15000
Rank in Ordered Dataset
i
Fischer G2M
-2.92
2.15
j
Diapause Score
-0.305
0.00904
k
Diapause score
0.0
−0.2
−0.4
−0.6
Treatment
Control
Short tx
Long tx
DND-41
LNCaP
MDA-MB-231
PC9
SK-MEL-28
Cell line

Figure 4: TMCI identifies distinct transcriptional programs across short-term and long-term treated drug-tolerant persister cells across cancer types. **a** Counts of T-cell acute lymphoblastic leukemia (DND-41), pancreatic (LNCaP), melanoma (SK-MEL-28), lung (PC9), and breast (MDA-MB-231) cancer cells from public persister-cell scRNA-seq experiments. Each cell line (control) received a short- (1-3 days) or long-term (6-7 weeks) anti-cancer treatment. **b,c** TMCI tree of cells from **a** colored by cell line and treatment condition (**b**) or by average expression of *ID2* per node (**c**). **d** Box-and-whisker plot of *ID2* expression for cells from each treatment condition. **e** The top 10 enriched pathways determined by Metascape[40] of the top 100 upregulated genes among short-term (top) and long-term (bottom) treated cells. Pathways relevant to the regulation of cellular proliferation are highlighted in red. **f,g** Normalized enrichment scores (NES) and respective *q*-values from gene set enrichment analysis (GSEA)[41] of short-term (**f**) and long-term (**g**) treated cells compared to corresponding control cells. "FISCHER_G2_M_CELL_CYCLE" is highlighted in red. **h** GSEA curve of the "FISCHER_G2_M_CELL_CYCLE" gene set for short-term treated cells against untreated cells. **i** NES scores of the "FISCHER_G2_M_CELL_CYCLE" gene set for each node against all other nodes in the TMCI tree from (**b**). **j** TMCI tree from **b** colored by diapause gene signature. **k** Box-and-whisker of diapause signature scores for cells from each treatment condition. For all box-and-whisker plots, center line: median, box bounds: interquartile range, whiskers: minimum and maximum scores. Statistical annotations represent results of a one-sided Mann-Whitney *U* test with Benjamini–Hochberg correction. ***: $p < 0.001$, ****: $p < 0.0001$, ns: not significant.

Table 1: Human cancer cell lines from single-cell RNA-sequencing persister-cell experiments used in this case study. Corresponding anti-cancer drugs, treatment duration, and GEO accession numbers are listed.

**Disease area**	**Cell line**	**Treatment**	**Duration**	**GEO Accession**
Prostate cancer	LNCaP	DMSO	48h	GSM5155455
		Enzalutamide	48h	GSM5155456
Melanoma	SK-MEL-28	Untreated		GSM4932163
		Dabrafenib	72h	GSM4932166
Non-small cell lung cancer	PC9	Untreated		GSM3972651
		Erlotinib	72h	GSM3972652
Breast cancer	MDA-MB-231	Untreated		GSM4684556
		Doxorubicin	7w	GSM4684557
T-cell acute lymphoblastic leukemia	DND-41	DMSO	24h	GSM4121361
		Compound E	24h	GSM4121362
		Compound E	6w	GSM4121364

Click here to access/download
Supplementary Material
supplementary.pdf

Click here to access/download
Supplementary Material
table_s1_mean_time.csv

Click here to access/download
Supplementary Material
table_s2_stats_time.csv

Click here to access/download
Supplementary Material
table_s3_mean_memory.csv

Click here to access/download
Supplementary Material
table_s4_stats_memory.csv

Click here to access/download

Supplementary Material

table_s8_mdamb231_log2fc.csv

Click here to access/download
Supplementary Material
table_s9_mdamb231_gsea.csv

Click here to access/download
Supplementary Material
table_s10_gsea_long.csv

Click here to access/download
Supplementary Material
table_s11_gsea_short.csv

Click here to access/download
Supplementary Material
table_s12_diapause_stats.csv

Click here to access/download
Supplementary Material
response_to_reviewers.pdf

Cover Letter

Click here to access/download;Personal Cover;cover_letter_rebuttal_1.pdf

UHN Princess Margaret Cancer Centre

Gregory W. Schwartz, Ph.D.
101 College Street, Room 11-308
Princess Margaret Cancer Centre
Toronto, ON Canada, M5G 1L7
gregory.schwartz@uhn.ca

May 22, 2024

Dear Hans Zauner,

Thank you for the opportunity to re-submit our manuscript. We believe that we have successfully addressed all comments raised by the referees from our initial submission. The reviewers' suggestions have significantly strengthened our manuscript without changing the overall story. Our work, which introduces a new interactive interface for single-cell data visualization, will greatly further our ability to explore the heterogeneity of -omics data sets.

The main overall comments were as follows. Reviewer 1 requested (1) interoperability with existing single-cell frameworks, (2) clarification of compatability with other normalization techniques, (3) exploration of fine-grain nodes within the tree. Reviewer 2 requested (4) additional interface features and documentation of code. Reviewer 3 requested (5) Tabula Sapiens for benchmarking, (6) visualization comparisons with updated colors, and (7) language updates.

To address these comments, we provided 24 new figure panels and included several new data including the following highlights:

1. Additional benchmarks using the large Tabula Sapiens data set including 483,152 cells. This analysis shows the efficient performance of TMCI on extremely large data sets.
2. A Batch-effect correction analysis showing different ways of using TMCI on non-count matrix data.
3. Cell-cycle analyses, showing that no single branch in the TMCI tree is dominated by a specific step in the cell cycle.
4. A new python implementation of TooManyCells that works with Scanpy and exports directly to TMCI, along with additional scripts converting Seurat/SingleCellExperiment data structures for use with TMCI.
5. A new feature of TMCI with average-weighted color blending of an arbitrary number of individual genes.

Over the course of the last three months, we have made a considerable effort to address the reviewer's comments. With these additional results, we believe that our revised manuscript has **addressed all reviewer suggestions**. Please find attached the completed detailed list of additions and changes in our point-to-point response. Major changes are highlighted in orange.

Sincerely,

Gregory Schwartz

Scientist, Princess Margaret Cancer Centre, University Health Network
Assistant Professor, Department of Medical Biophysics, University of Toronto
Faculty Affiliate, Vector Institute
