# Supplementary material for: TooManyCellsInteractive: A visualization tool for dynamic exploration of single-cell data
Source: Gigascience. 2024 Aug 22;13:giae056. doi: 10.1093/gigascience/giae056 (PMC11340645; doi:10.1093/gigascience/giae056)
Supplement: giae056_Supplemental_Files [file giae056_supplemental_files.zip › figure_s3_comparison.pdf]

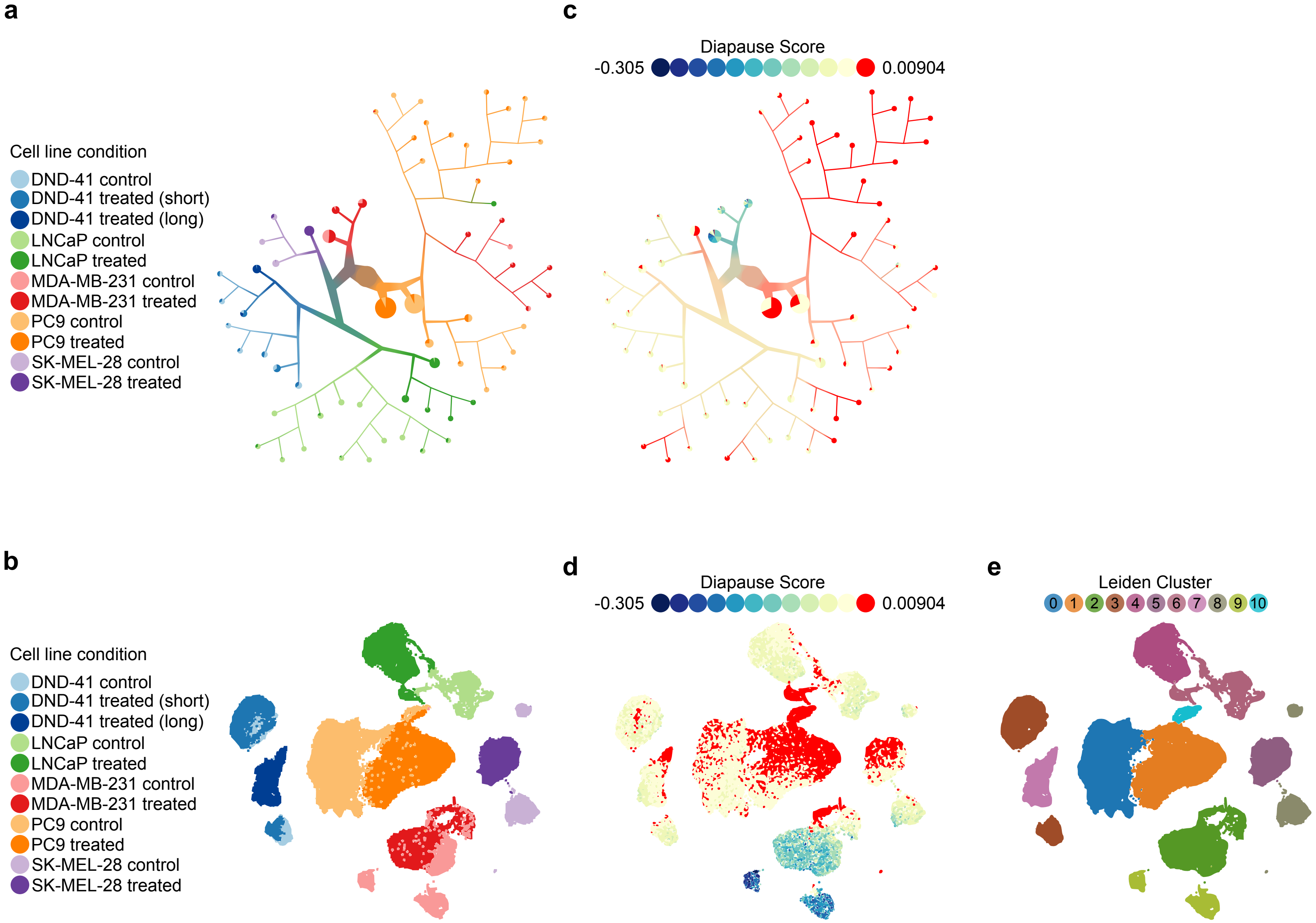
a
Cell line condition
DND-41 control
DND-41 treated (short)
DND-41 treated (long)
LNCaP control
LNCaP treated
MDA-MB-231 control
MDA-MB-231 treated
PC9 control
PC9 treated
SK-MEL-28 control
SK-MEL-28 treated
c
Diapause Score
-0.305
0.00904
b
Cell line condition
DND-41 control
DND-41 treated (short)
DND-41 treated (long)
LNCaP control
LNCaP treated
MDA-MB-231 control
MDA-MB-231 treated
PC9 control
PC9 treated
SK-MEL-28 control
SK-MEL-28 treated
d
Diapause Score
-0.305
0.00904
e
Leiden Cluster
0 1 2 3 4 5 6 7 8 9 10
