# Supplementary material for: TooManyCellsInteractive: A visualization tool for dynamic exploration of single-cell data
Source: Gigascience. 2024 Aug 22;13:giae056. doi: 10.1093/gigascience/giae056 (PMC11340645; doi:10.1093/gigascience/giae056)
Supplement: giae056_Supplemental_Files [file giae056_supplemental_files.zip › figure_s4_harmony.pdf]

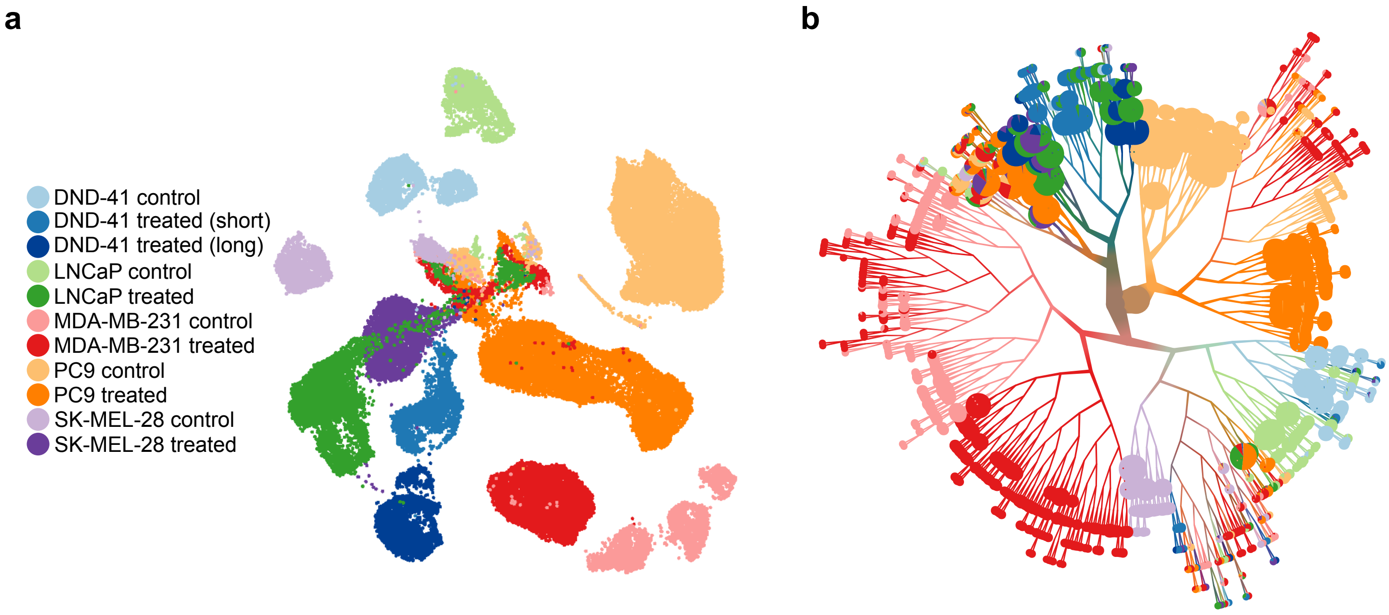
a
b
DND-41 control
DND-41 treated (short)
DND-41 treated (long)
LNCaP control
LNCaP treated
MDA-MB-231 control
MDA-MB-231 treated
PC9 control
PC9 treated
SK-MEL-28 control
SK-MEL-28 treated
