# Supplementary figures and images for: TooManyCellsInteractive: A visualization tool for dynamic exploration of single-cell data
Source: Gigascience. 2024 Aug 22;13:giae056. doi: 10.1093/gigascience/giae056 (PMC11340645; doi:10.1093/gigascience/giae056)

**a**

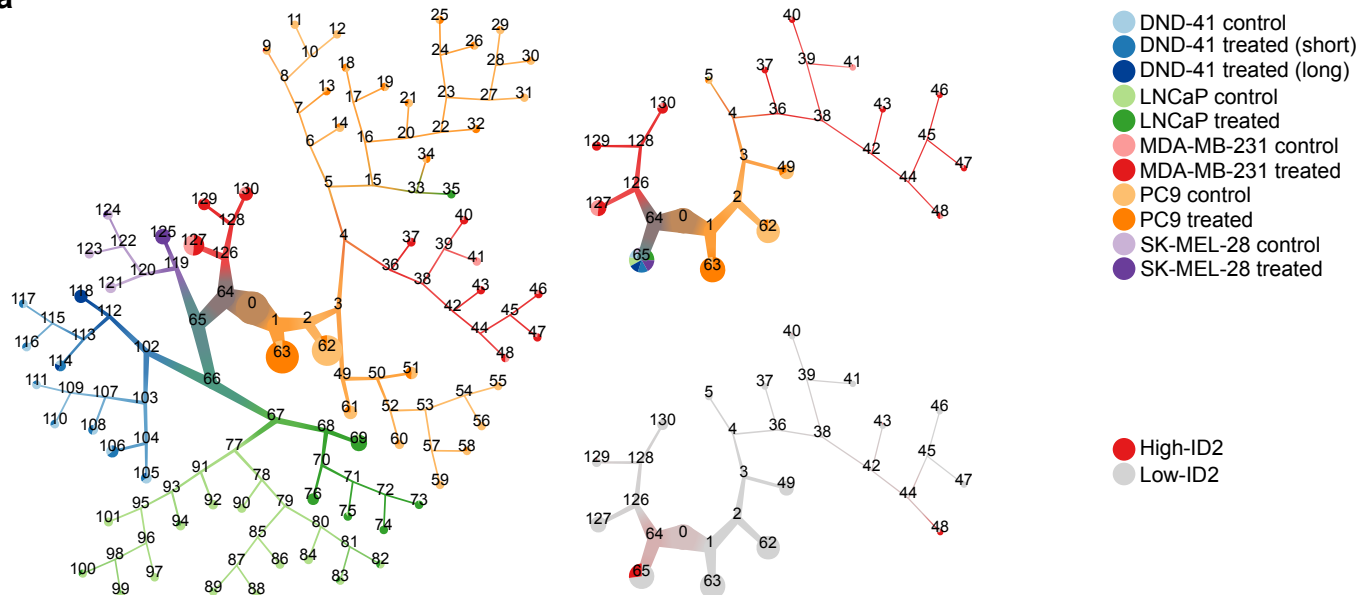

**b**

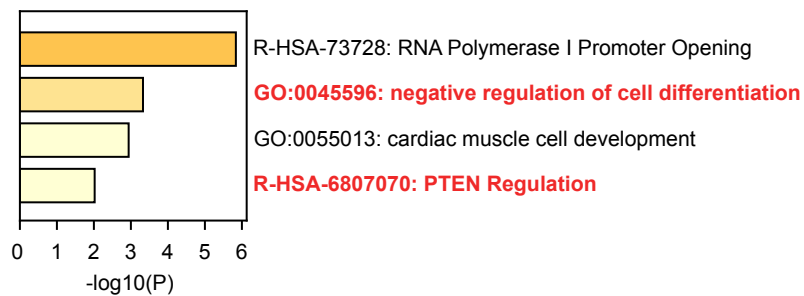

**c**

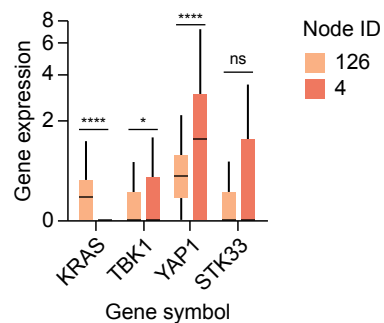

Supplement: giae056_Supplemental_Files [file giae056_supplemental_files.zip › figure_s1_mdamb231.pdf]

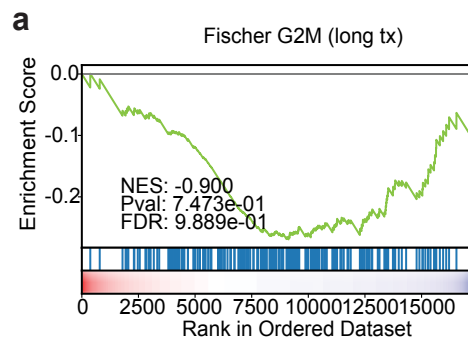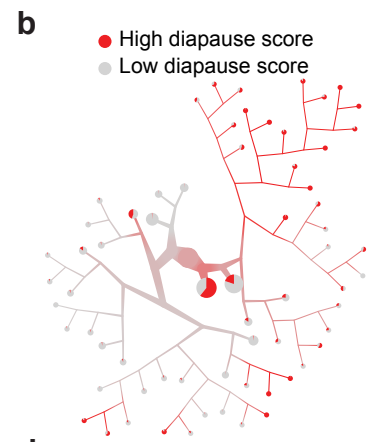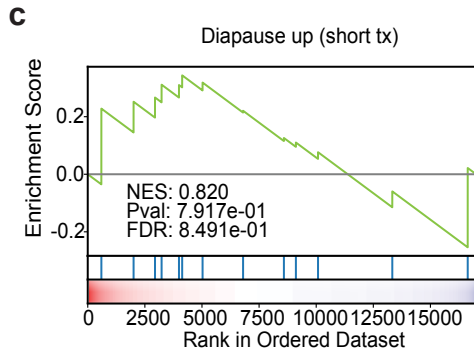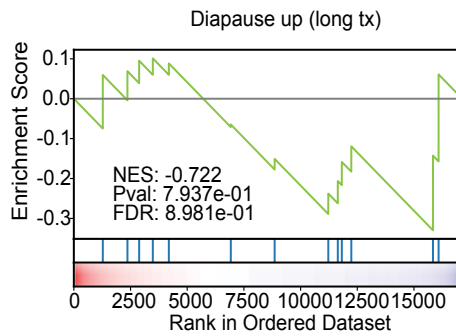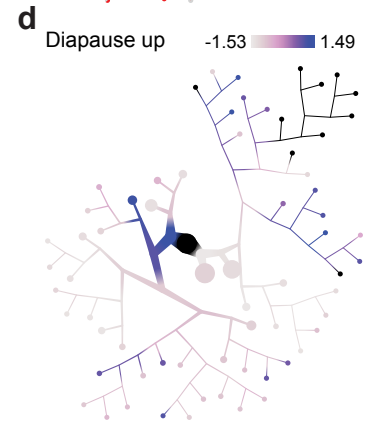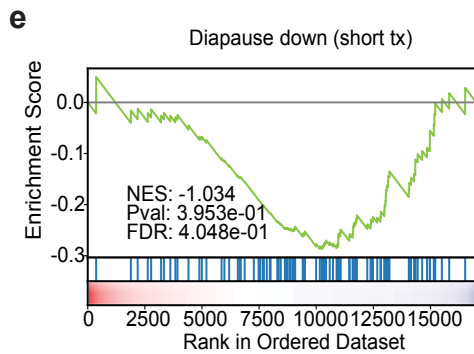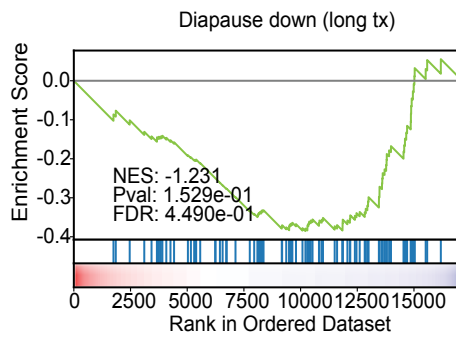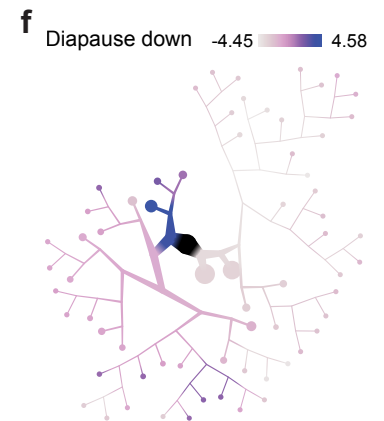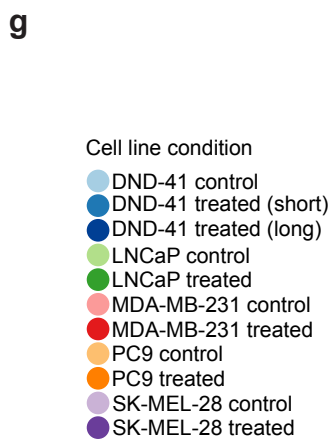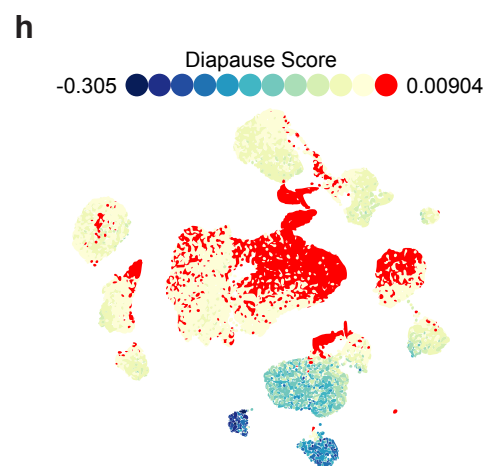

Supplement: giae056_Supplemental_Files [file giae056_supplemental_files.zip › figure_s2_cell_lines_treated.pdf]
