# Supplementary material for: TooManyCellsInteractive: A visualization tool for dynamic exploration of single-cell data
Source: Gigascience. 2024 Aug 22;13:giae056. doi: 10.1093/gigascience/giae056 (PMC11340645; doi:10.1093/gigascience/giae056)
Supplement: giae056_Response_to_Reviewer_Comments_Original_Submission [file giae056_response_to_reviewer_comments_original_submission.pdf]

Author's Response To Reviewer Comments

Please see `response_to_reviewers.pdf` for a complete point-by-point response.
