# Supplementary material for: TooManyCellsInteractive: A visualization tool for dynamic exploration of single-cell data
Source: Gigascience. 2024 Aug 22;13:giae056. doi: 10.1093/gigascience/giae056 (PMC11340645; doi:10.1093/gigascience/giae056)
Supplement: giae056_Response_to_Reviewer_Comments_Revision_1 [file giae056_response_to_reviewer_comments_revision_1.pdf]

Author's Response To Reviewer Comments

Dear Hans Zauner,

Thank you for accepting our manuscript. We have made the following changes as requested:

1. We added the reference to the bibliography section with the requested link, cited in the code availability section.

2. We added ORCIDs to all authors in the title page.

3. We removed all highlighting.

4. We added all required files to build the LaTeX document using `latexmk -lualatex manuscript.tex`.

Please let me know if anything else is required.

Sincerely,

Gregory Schwartz
