# Supplementary material for: TooManyCellsInteractive: A visualization tool for dynamic exploration of single-cell data
Source: Gigascience. 2024 Aug 22;13:giae056. doi: 10.1093/gigascience/giae056 (PMC11340645; doi:10.1093/gigascience/giae056)
Supplement: giae056_Reviewer_1_Report_Original_Submission — Qingnan Liang, Ph.D. -- 1/26/2024 Reviewed [file giae056_reviewer_1_report_original_submission.pdf]

Reviewer Report

Title: TooManyCellsInteractive: a visualization tool for dynamic exploration of single-cell data

Version: Original Submission **Date:** 1/26/2024

Reviewer name: Qingnan Liang, Ph.D.

Reviewer Comments to Author:

Klamann et al. report a tool for single-cell data visualization, TMCI, which was related to the previous method of TMC. It is appreciated to see such continuous work and maintenance of the method and I do agree TMCI has the potential of promoting the application of TMC. The manuscript is generally well-written, and it suits well with the scope of GigaScience. The TMCI is publicly available with reasonably detailed tutorials. In this manuscript, however, at several points the elaboration does not provide sufficient details or rationales. I suggest revision/clarification as below before recommendation to publish.1. Does TMCI provide an interface with one or more popular single-cell frameworks, such as SingelCellExperiment, Seurat, or Scanpy? A TMCI user would probably use one of these frameworks to do other parts of the analysis.2. Is batch effect considered in the drug-treated data example? More generally, if a user want to use TMCI with multiple datasets, what would be the recommended approach for batch effect? Also, we know cell cycle is a factor that are usually 'regressed out' for single-cell analysis. Does TMC/TMCI consider this?3. "To normalize cells between data sets, we used term frequency-inverse document frequency to weigh genes such that more frequent genes across cells had less impact on downstream clustering analyses" We know TF-IDF is becoming a common practice in scATAC-seq analysis. Is this TF-IDF approach common for tree construction (or hierarchical clustering) with high dimensional data? Is this recommended for all users with scRNA-seq data?4. Figure 4C is not very easy to read. It may be helpful to label/highlight the comparison pairs to make the point.5. Also it is not sufficiently emphasized that how TMCI helped finding this ID2 target. Or how such visualization would trigger interesting downstream approaches. I guess the power of this tree approach is somehow similar to the increasingly popular 'metacell' approach, which combine similar cells to 'cell states'. Thus it makes an interesting midpoint between 'single-cell' and 'pseudo-bulk'. It would really be helpful to see that some states (nodes), although similarly treated, behave differently than others, if there are such cases (not sure if cell lines have such heterogeneity). Similar comments for the pathway analysis part.

Level of Interest

Please indicate how interesting you found the manuscript: Choose an item.

Quality of Written English

Please indicate the quality of language in the manuscript: Choose an item.

Declaration of Competing Interests

Please complete a declaration of competing interests, considering the following questions:

- Have you in the past five years received reimbursements, fees, funding, or salary from an organisation that may in any way gain or lose financially from the publication of this manuscript, either now or in the future?
- Do you hold any stocks or shares in an organisation that may in any way gain or lose financially from the publication of this manuscript, either now or in the future?
- Do you hold or are you currently applying for any patents relating to the content of the manuscript?
- Have you received reimbursements, fees, funding, or salary from an organization that holds or has applied for patents relating to the content of the manuscript?
- Do you have any other financial competing interests?
- Do you have any non-financial competing interests in relation to this paper?

If you can answer no to all of the above, write 'I declare that I have no competing interests' below. If your reply is yes to any, please give details below.

I declare that I have no competing interests

I agree to the open peer review policy of the journal. I understand that my name will be included on my report to the authors and, if the manuscript is accepted for publication, my named report including any attachments I upload will be posted on the website along with the authors' responses. I agree for my report to be made available under an Open Access Creative Commons CC-BY license (http://creativecommons.org/licenses/by/4.0/). I understand that any comments which I do not wish to be included in my named report can be included as confidential comments to the editors, which will not be published.

Choose an item.

To further support our reviewers, we have joined with Publons, where you can gain additional credit to further highlight your hard work (see: https://publons.com/journal/530/gigascience). On publication of this paper, your review will be automatically added to Publons, you can then choose whether or not to claim your Publons credit. I understand this statement.

Yes Choose an item.
