# Supplementary material for: TooManyCellsInteractive: A visualization tool for dynamic exploration of single-cell data
Source: Gigascience. 2024 Aug 22;13:giae056. doi: 10.1093/gigascience/giae056 (PMC11340645; doi:10.1093/gigascience/giae056)
Supplement: giae056_Reviewer_2_Report_Original_Submission — Mehmet Tekman -- 2/12/2024 Reviewed [file giae056_reviewer_2_report_original_submission.pdf]

Reviewer Report

Title: TooManyCellsInteractive: a visualization tool for dynamic exploration of single-cell data

Version: Original Submission **Date:** 2/12/2024

Reviewer name: Mehmet Tekman

Reviewer Comments to Author:

______________________________PAPER: TOOMANYCELLSINTERACTIVE REVIEW______________________________Table of Contents__________________1. Using the Application.. 1. Positive Notes:..... 1. General UI and Execution.. 2. Negative Notes:..... 1. Controls..... 2. Documentation..... 3. Feature Overlays:2. Docker / Postgreseql3. Ethos of the IntroductionThe manuscript reads very well, and the quality of the language isgood.This review tests the application itself, and makes some comment aboutsome ambiguous wording in the introduction1 Using the Application======================I tested the Interactive Display at <https://tmci.schwartzlab.ca/>1.1 Positive Notes:~~~~~~~~~~~~~~~~~~1.1.1 General UI and Execution------------------------------The general interactivity of the UI was very impressive andexpressive. I liked that every aspect including the pies and the linesthemselves could be coloured and scaled.I found the feature overlays and pruning history stack very intuitive,as well as rolling back the history on each state change.The choice of D3 was a good one, enabling very pleasing animationsenter/exit/update state animations, as well as ease of SVG export.The inclusion of a command line `generate-svg.sh' for renderingwithout a browser is very useful.1.2 Negative Notes:~~~~~~~~~~~~~~~~~~1.2.1 Controls--------------At first I wasn't able to find the controls, despite having the pageopen to 1330px wide, but then I realised I had to scroll down outsideof the SVG container to find them.As mentioned in a recently opened PR, there's a CSS media rule `@mediaonly screen and (min-width:1238px)' taking place, that looks strangeon my Firefox 122 on Linux. Maybe better media rules for screens inthe 700-900px wide range might be useful, as well as making separaterules for smartphones.1.2.2 Documentation-------------------Typescript is a good language to develop in, and lends itselfnaturally to documentation, though I did notice a distinct lack ofdocumentation above many functions in the code base.Perhaps write a bit more documentation to make the code baseaccessible to new collaborators?Otherwise, the quality of code looked good, and the license was GPLv3which is always welcome.1.2.3 Feature Overlays:----------------------I found the feature overlays super useful, though limited by thenumber of colours. These appear to be limited to one colour for allgenes.Very useful for showing multiple genes, but it would be nice to havethe ability to colour the expression of different genes with differentcolours, at least for < 3 genes of interest (due to the difficultcolour mixing constraints).2 Docker / Postgreseql=====================It is not clear to me what the Node server and PostgresQL database runin the docker container are actually doing, other than fetching cellmetadata and marking user subsets from pruning actions.Could this not have been implemented in Javascript (e.g. IndexedDB)?Why does the data need to be hosted, if it's the user loading it fromtheir own machine anyway. Is the idea that the visualization should beshared by multiple users who will be accessing the same dataset?If this is a single-user analysis, then why not keep all thecomputation and retrieval on the

client-side?The reason I'm asking this is because I believe that by keeping thedatabase operations within Javascript, you could run the system withina single Conda environment, or even with pure Node lockfile.I can understand needing a Docker for development purposes, but toactually run the software itself seems excessive. Is it not possibleto separate the client and server into Conda? That way, one could theninclude the vizualisation (as the end stage) in bioinformaticpipelines.3 Ethos of the Introduction==========================This is a small wording complaint in the Introduction section.TooManyCellsInteractive (TMCI) presents itself as a solution to theconventional scRNA-seq workflows that prepare the data via the usual:data â†' PCA â†' UMAPâ†' kNN â†' clustering stages.TMCI hints that it as an alternative solution to this workflow, butfrom what I can see in the documentation, it appears to require a`cluster_tree.json' file, one that is produced only by theTooManyCells (TMC) pipeline.Unless I've misunderstood, it's not accurate to say that TMCI is analternative to these conventional workflows, but that TMC is.TMCI simply consumes the files output by TMC and renders them. If whatI'm saying is true, then the introduction should reflect that.

Level of Interest

Please indicate how interesting you found the manuscript: Choose an item.

Quality of Written English

Please indicate the quality of language in the manuscript: Choose an item.

Declaration of Competing Interests

Please complete a declaration of competing interests, considering the following questions:

- Have you in the past five years received reimbursements, fees, funding, or salary from an organisation that may in any way gain or lose financially from the publication of this manuscript, either now or in the future?
- Do you hold any stocks or shares in an organisation that may in any way gain or lose financially from the publication of this manuscript, either now or in the future?
- Do you hold or are you currently applying for any patents relating to the content of the manuscript?
- Have you received reimbursements, fees, funding, or salary from an organization that holds or has applied for patents relating to the content of the manuscript?
- Do you have any other financial competing interests?
- Do you have any non-financial competing interests in relation to this paper?

If you can answer no to all of the above, write 'I declare that I have no competing interests' below. If your reply is yes to any, please give details below.

I declare that I have no competing interests

I agree to the open peer review policy of the journal. I understand that my name will be included on my report to the authors and, if the manuscript is accepted for publication, my named report including any attachments I upload will be posted on the website along with the authors' responses. I agree for my report to be made available under an Open Access Creative Commons CC-BY license (http://creativecommons.org/licenses/by/4.0/). I understand that any comments which I do not wish to be included in my named report can be included as confidential comments to the editors, which will not be published.

Choose an item.

To further support our reviewers, we have joined with Publons, where you can gain additional credit to further highlight your hard work (see: https://publons.com/journal/530/gigascience). On publication of this paper, your review will be automatically added to Publons, you can then choose whether or not to claim your Publons credit. I understand this statement.

Yes Choose an item.
