# Supplementary material for: TooManyCellsInteractive: A visualization tool for dynamic exploration of single-cell data
Source: Gigascience. 2024 Aug 22;13:giae056. doi: 10.1093/gigascience/giae056 (PMC11340645; doi:10.1093/gigascience/giae056)
Supplement: giae056_Reviewer_3_Report_Original_Submission — Georgios Fotakis -- 2/19/2024 Reviewed [file giae056_reviewer_3_report_original_submission.pdf]

Reviewer Report

Title: TooManyCellsInteractive: a visualization tool for dynamic exploration of single-cell data

Version: Original Submission **Date:** 2/19/2024

Reviewer name: Georgios Fotakis

Reviewer Comments to Author:

1) General commentsIn this manuscript the authors present TooManyCellsInteractive (TMCI), a browser-based TypeScript graphical user interface for the visualization and interactive exploration of single-cell data. TMCI facilitates the visualization of single-cell data by representing it as a radial tree of nested cell clusters. It relies on TooManyCells, a suite of tools designed for multi-resolution and multifaceted exploration of single-cell clades based on a matrix-free divisive hierarchical spectral clustering method. A key advantage of TCMI lies in its capability to provide a quantitative depiction of relationships among clusters, allowing for the delineation of context-dependent rare and abundant cell populations, as showcased in the original publication [1] and in the present manuscript. TMCI extends the capabilities of TMC significantly, notably enhancing computational performance, particularly in scenarios where multiple features are overlaid (an improvement that is attributed to the persistent feature of the PostgreSQL database).A notable aspect of this manuscript is the fact that the authors performed a benchmark using publicly available scRNAseq datasets. This benchmark highlights TMCI's superior performance over TMC and its comparable performance to two other commonly utilized tools (Cirrocumulus and CELLxGENE). Moreover, the authors showcase TMCI's applicability through aggregating publicly available scRNAseq data. Here, they successfully delineate sub-populations of cancer drug-tolerant persister cells by employing minimum distance search pruning, enhancing the visibility of small sub-populations. Additionally, the authors note an increase in ID2 gene expression among persister-cell populations, as well as the enrichment of unique biological programs between short- and long-term persister-cell populations. Furthermore, they observe an upregulation of the diapause gene signature across all treated sub-populations. The biological insights the authors glean are novel and highly intriguing.In general, this manuscript is well written, with the authors offering comprehensive documentation that covers the essential steps for installing and running TMCI through their GitHub repository. Additionally, they provide a minimal dataset as an example for users. However, there are a few minor adjustments that, once implemented, would enhance the manuscript's value by improving clarity and providing valuable insights to the field.2) Specific comments for revisiona) Major-As stated in the manuscript's abstract, visualising large cell populations from single-cell atlases poses greater challenges and demands compute-intensive processes. One of my major concerns revolves around TMCI's scalability when handling large datasets. The authors conducted benchmarking on relatively modest datasets (ranging from 18,859 to 54,220 cells). Based on the data provided in Supplementary Table S3, while TMCI demonstrates comparable performance to CELLxGENE on the Tabula Muris dataset and its subset (with mean memory consumption differences ranging from 870 MB to 1.8 GB), the disparity significantly increases when loading and rendering visualizations of the larger dataset, reaching 8.5 GB of RAM. It would be of great interest if the authors conducted a similar

benchmark using a larger dataset to elucidate how TMCI scales with increased cell numbers, especially considering the trend in the field towards single-cell atlases and the availability of datasets consisting up to millions of cells (like the Tabula Sapiens [2] dataset or similar [3, 4]).- In the "Results" section, under the title "TMCI identifies sub-populations with highly expressed diapause programs," the authors assert that "the significantly different sub-populations were more easily seen in TMCI's tree". Since perception can be subjective (for instance, a user more accustomed to UMAP plots may find it challenging to interpret a tree representation), it would be beneficial for the authors to allocate a section of the supplementary material to demonstrate the clarity advantages of TMCI's tree visualization. One approach could involve a side-by-side comparison of visualizations generated by TMCI and CELLxGENE using the same color scheme. For instance, Figure 4b could be compared with Supplementary Figure S1g, Figure 4j with Supplementary Figure S1h, and so forth.- The "Discussion" section overlooks the future prospects of TMCI. As demonstrated in the case study, TMCI exhibits potential beyond serving as a visualization tool for identifying tree-based relationships in single-cell data. Are there any plans for integrating analytical functionalities to provide insights into cellular compositions and underlying biology, such as marker gene identification, differential gene expression analysis, and gene set enrichment analysis? In the future, could TMCI support the visualization of such results using methods like violin plots, heatmaps, and others?- In the "Materials and Methods'' section, the authors outline the process of aggregating the scRNAseq datasets used for the case study, including filtering and normalization steps. However, scRNAseq technologies are prone to significant noise resulting from amplification and dropout events. Additionally, when integrating different scRNAseq datasets, users need to consider potential batch effects. Did the authors employ any de-noising or batch correction methods? If not, what was the rationale behind this decision? It would be intriguing to observe any potential differences in the results following the application of such methods.- Remaining within the "Materials and Methods" section, providing a brief description of the methods and tools utilized for the differential gene expression analysis, the GSEA (if not solely conducted through Metascape), and the packages utilized to generate the plots in Figures 3 and 4 would enhance clarity and facilitate reproducibility.- Figure 4 - b: Distinguishing between the various cell lines on the partitioned nodes based on the current color codingâ€"particularly for the MDA-MB-231 and PC9 cell lines, as well as between the treated and untreated populations of the SK-MEL-28 cell lineâ€"is quite challenging. Employing a different color scheme would significantly enhance clarity, making the different cell populations more distinguishable.- Figure 4 - d and k: The authors should add statistics as relying solely on the box and whisker plots makes it challenging to ascertain whether there is a significant difference between the conditions. For instance, it appears that ID2 is over-expressed between the control and treated population only in the SK-MEL-28 cell line.b) Minor- In the "Results" section, under the title "TMCI reduces time to display trees," the authors state: "these benchmarks indicate not only the superior performance of TMCI to generate static and interactive tree of single-cell data compared to other tools…". However, based on the results presented in the manuscript and the supplementary material, it seems that TMCI may not be outperforming alternative interactive visualization methods. This phrase should be revised to accurately reflect the benchmark results.References1. Schwartz GW, Zhou Y, Petrovic J, Fasolino M, et al. TooManyCells identifies and visualizes relationships of single-cell clades. Nat Methods 2020;17(4):405-413. PMID: 321233972. The Tabula Sapiens Consortium, The Tabula Sapiens: A multiple-organ, single-cell transcriptomic atlas of humans. Science 2022;376,

eabl4896. DOI:10.1126/science.abl48963. Sikkema L, RamÃ-rez-SuÃ¡stegui C, Strobl DC, et al. An integrated cell atlas of the lung in health and disease. Nat Med 2023;29, 1563-1577. DOI:10.1038/s41591-023-02327-24. Salcher S, Sturm G, Horvath L, et al. High-resolution single-cell atlas reveals diversity and plasticity of tissue-resident neutrophils in non-small cell lung cancer. Cancer cell 2022;40(12):1503-1520.E8. DOI:10.1016/j.ccell.2022.10.008

Level of Interest

Please indicate how interesting you found the manuscript: Choose an item.

Quality of Written English

Please indicate the quality of language in the manuscript: Choose an item.

Declaration of Competing Interests

Please complete a declaration of competing interests, considering the following questions:

- Have you in the past five years received reimbursements, fees, funding, or salary from an organisation that may in any way gain or lose financially from the publication of this manuscript, either now or in the future?
- Do you hold any stocks or shares in an organisation that may in any way gain or lose financially from the publication of this manuscript, either now or in the future?
- Do you hold or are you currently applying for any patents relating to the content of the manuscript?
- Have you received reimbursements, fees, funding, or salary from an organization that holds or has applied for patents relating to the content of the manuscript?
- Do you have any other financial competing interests?
- Do you have any non-financial competing interests in relation to this paper?

If you can answer no to all of the above, write 'I declare that I have no competing interests' below. If your reply is yes to any, please give details below.

I declare that I have no competing interests

I agree to the open peer review policy of the journal. I understand that my name will be included on my report to the authors and, if the manuscript is accepted for publication, my named report including any attachments I upload will be posted on the website along with the authors' responses. I agree for my report to be made available under an Open Access Creative Commons CC-BY license (http://creativecommons.org/licenses/by/4.0/). I understand that any comments which I do not wish to be included in my named report can be included as confidential comments to the editors, which will not be published.

Choose an item.

To further support our reviewers, we have joined with Publons, where you can gain additional credit to further highlight your hard work (see: https://publons.com/journal/530/gigascience). On publication of this paper, your review will be automatically added to Publons, you can then choose whether or not to claim your Publons credit. I understand this statement.

Yes Choose an item.
